# Drugs to limit Zika virus infection and implication for maternal-fetal health

**DOI:** 10.3389/fviro.2022.928599

**Published:** 2022-08-05

**Authors:** Ankur Kumar, Deepak Kumar, Joyce Jose, Rajanish Giri, Indira U. Mysorekar

**Affiliations:** 1 Department of Medicine, Section of Infectious Diseases, Baylor College of Medicine, Houston, TX, United States; 2 School of Basic Sciences, Indian Institute of Technology Mandi, VPO-Kamand, Mandi, India; 3 Department of Biochemistry and Molecular Biology, The Pennsylvania State University, University Park, PA, State College, United States; 4 Department of Molecular Virology and Microbiology, Baylor College of Medicine, Houston, TX, United States

**Keywords:** pregnancy, placenta, vertical transmission, microcephaly, NS2B protease, NS3, drug targets

## Abstract

Although the placenta has robust defense mechanisms that protect the fetus from a viral infection, some viruses can manipulate or evade these mechanisms and disrupt physiology or cross the placental barrier. It is well established that the Zika virus is capable of vertical transmission from mother to fetus and can cause malformation of the fetal central nervous system (i.e., microcephaly), as well as Guillain-Barre syndrome in adults. This review seeks to gather and assess the contributions of translational research associated with Zika virus infection, including maternal-fetal vertical transmission of the virus. Nearly 200 inhibitors that have been evaluated *in vivo* and/or *in vitro* for their therapeutic properties against the Zika virus are summarized in this review. We also review the status of current vaccine candidates. Our main objective is to provide clinically relevant information that can guide future research directions and strategies for optimized treatment and preventive care of infections caused by Zika virus or similar pathogens.

## Introduction

The Zika virus (ZIKV) was first isolated from a rhesus monkey in 1947 in the Zika forest during the surveillance of yellow fever disease in Uganda. Only 14 human cases of ZIKV had been confirmed prior to 2007, when 49 confirmed cases and 59 probable cases of infection occurred in the Yap Islands of the Federated States of Micronesia, marking the first known ZIKV outbreak (1, 2). Larger outbreaks followed in French Polynesia (3, 4) and elsewhere in the Pacific (5), eventually reaching Brazil by 2015 (6, 7). In early December of 2015, the Pan American Health Organization (PAHO) and the World Health Organization (WHO) reported a possible association between ZIKV infection and sudden increase in microcephaly and Guillain-Barre syndrome cases (8), leading the WHO to declare a Public Health Emergency of International Concern (PHEIC) for ZIKV in 2016 (9). An estimated 700,000 individuals in the Americas were infected with ZIKV by 2016. Although the ZIKV epidemic has since subsided, the virus continues to circulate in the Americas and other regions of the world (10, 11). As of 2019, active transmission has been reported globally in 87 countries and territories (12). And in 2021, an outbreak of at least 231 cases was reported in a region of southwest India where ZIKV had not been seen previously (13, 14).

Transmission of ZIKV occurs primarily through *Aedes* mosquitoes that are native to Asia and Africa but have spread globally (15, 16). ZIKV is the first flavivirus known to be transmitted from human to human through sex (17-20) and transmission has also been documented through blood transfusion (21-24). Evidence of vertical transmission of the ZIKV from mother to fetus across the placental barrier was first reported in French Polynesia cases from 2013-14 (25). Additional evidence established this as a mode of transmission emerged during the Brazil epidemic of 2015-16, as the presence of ZIKV antigen and RNA was identified in placental tissue and amniotic fluids of women infected with the virus and also in the brain tissue from fetuses and newborn babies with microcephaly (who were deceased following birth) (26-29).

These events highlight the additional risks that pregnant women face when infected with a pathogen due to outcomes that may affect the health of the fetus. Birth defects, congenital disabilities, and a host of other serious complications have been associated with infections of several pathogens during pregnancy (30, 31). Although the placenta is a remarkably sophisticated barrier that protects the fetus during most maternal infections, some pathogens can cross the placental barrier and reach the fetus by evading or manipulating placental defense mechanisms. Such pathogens, including toxoplasma gondii, rubella virus, cytomegalovirus (CMV), and herpes simplex virus (commonly referred to as TORCH pathogens in clinics), can result in vertical transmission from mother to the fetus (32). These perinatal infections contribute to 2-3% of the overall congenital anomalies, including disabilities of the neurological or cardiovascular system and, in some cases, even the death of the fetus (33). Because it is now established that ZIKV infection during pregnancy is associated with malformation of the fetal central nervous system and can result in microcephaly (34), it has been reasonably suggested that the ZIKV be added to the group of TORCH pathogens that are designated to be of significant concern to pregnant women (35).

## ZIKV-Induced microcephaly and associated mechanisms

The placenta acts as a protective barrier against most pathogens. However during a ZIKV infection, it can instead serve as a mediator for the transmission of the virus from mother to fetus. The virus can cause damage to the human placenta during infection, leading to a condition known as chronic placentitis (28), in which the virus targets macrophages (36) and trophoblast cells (37). Findings from mouse model experiments suggest that infection during pregnancy *via* a subcutaneous route causes damage to the placenta and fetal demise (38) *via* an intravenous route that restricts intrauterine growth and *via* intraperitoneal injection that leads the infection to the fetal brain (39). Infection also induces apoptosis of the trophoblast cells and vascular damage that disrupts the placental barrier allowing the direct passage of ZIKV to the fetus (38). ZIKV replicates in the host by overcoming the interferon (IFN)-mediated host immune response that induces the degradation of the interferon-regulated transcriptional activator STAT2 (40, 41), leading to impairment of IFN induction and downstream IFN stimulated genes (41).

Previously, microcephaly and other neurological abnormalities have been associated with maternal-fetal vertical transmission of TORCH-designated pathogens, including CMV, the rubella virus, and herpes simplex virus (35). Association of Guillain-Barre syndrome with ZIKV was first reported in 2013-2014 during the French Polynesia outbreak (4, 42). Following the continued spread of ZIKV in Brazil and nearby territories (6, 43), an increase was observed in newborn cases of microcephaly, and association of these cases with ZIKV was suspected due to the documented presence of the virus in amniotic fluids and fetal tissues (34, 44). In 2016, the United States Centers for Disease Control and Prevention (CDC) reported that ~10% (24/250) of pregnancies with laboratory-confirmed ZIKV infection in the U.S. resulted in a fetus or newborn with infection-related birth defects (45).

Microcephaly can generally be classified as either congenital (present from birth) or postnatal (developed in the first two years) (46-49). Congenital microcephaly can result from aggressive environmental factors during intrauterine brain development, including the vertical transmission of viral infections, exposure to toxic substances or radiation, or nutritional deficiency (46, 47, 50). Biological factors such as chromosomal abnormalities and the impairment of a specific gene’s expression also influence the development of congenital microcephaly (48, 51-53). Postnatal microcephaly is an acquired condition that can result from external environmental factors such as brain injury, encephalitis, hemorrhage, and malnutrition, or may also occur as the result of several genetic disorders that affect brain development (46, 49).

After ZIKV-associated microcephaly was first reported in Brazil in 2016, further investigation established the tropism of the virus to human neural precursor cells (NPCs) and found evidence that the virus induces apoptosis, cell cycle perturbation, and defects in the differentiation process in the developing nervous system (54-57). Studies using a modeled culture system for blood-brain barrier (BBB) in adult mice revealed that ZIKV could penetrate the BBB, although the complete mechanism is unclear (58, 59). In microcephaly-associated tissues, many genes have been identified that are downregulated during ZIKV infection (55, 60, 61). ZIKV infection perturbs the cell cycle that regulates these genes, which decreases their expression levels (54, 60). Many of these genes encode proteins that are localized at the centrosome and have essential roles in cell cycle regulation (62), suggesting a molecular level mechanistic association between ZIKV infection and the progression of microcephaly. The cell cycle dysregulation caused by ZIKV can also lead to impaired neurogenesis (63). The *in vitro* experimental evidence suggests that this impaired neurogenesis might be due to impaired gene expression and mitosis (64, 65). The mitotic dysfunction increases the chance of cell cycle defects, chromosomal abnormalities, impaired NPC proliferation, and even cell death (65, 66).

Cell death contributes to ZIKV-induced microcephaly detected in the infected human fetal brains (26, 34). Experimental evidence in ZIKV-infected human NPCs reveals that the genes associated with apoptosis-related pathways are upregulated (60, 61). ZIKV-induced apoptosis may also be due to the activation of the immune response, leading to the upregulation of viral response genes associated with toll-like receptor (TLR) signaling, IFN signaling, and TNFα signaling pathways (61). In addition to the damage to the NPC, ZIKV infection can also cause axonal damage, gliosis, calcifications in the cortical plate, and microglial nodules, contributing to microcephaly phenotypes (26, 29, 34). Glial cells, microglia, and astrocytes are reported to be the primary targets of ZIKV infection (28, 67-69). Strikingly, ZIKV entry of ZIKV into glial cells is facilitated by AXL receptor expressed on the cell surface (68), resulting in the activation of AXL kinase activity, which further induces oxidative stress and triggers the innate immune response and inflammation, and upregulates the expression of TLRs, RIGI-like receptor (RLR) and NOD-like receptors (NLRs) (67, 68, 70). In contrast, imbalanced inflammation is associated with microcephaly and other neurological abnormalities related (and unrelated) to ZIKV infection (71-74). Thus, anti-inflammatory drugs might help reduce the neurological complications that arise in congenital Zika syndrome (75, 76).

## Structure, function, and genome

### ZIKV structure

The *Flaviviridae* family includes ZIKV, which shares many physical characteristics with other flaviviruses such as the dengue virus (DENV) and West Nile virus (WNV). The flavivirus particle exists as an immature non-infectious particle and a mature infectious particle (77). Initially, the virus particle assembles at the endoplasmic reticulum in its “spiky” non-infectious state. Afterward, the maturation of the virus particle occurs in the late Golgi, where low pH-mediated conformational changes take place in the viral surface glycoproteins, and the host furin protease cleaves the precursor membrane (prM) protein into the pr peptide and mature membrane (M) protein. The mature flavivirus surface comprises 180 copies each of envelope (E) and membrane (M) proteins, which exist as E-M heterodimers arranged in icosahedral symmetry. The E protein consists of domains I, II, and III and stem transmembrane domains (78). The M protein has a loop at the N terminus, stem, and transmembrane regions (79). The prM cleavage exposes the fusion loop on the E protein, which mediates the endosomal fusion at low pH during entry (80). ZIKV RNA is synthesized within the virus-induced replication vesicles derived from the modified endoplasmic reticulum of the host (78, 81, 82).

The first cryo-EM solved structure for a mature ZIKV (H/PF/2013 strain) at 37 °C was reported with an atomic resolution of 3.8 Å in 2016 (PDB ID: 5IRE) (79). A thermally stable virus structure was reported later the same year with a resolution of 3.7 Å at 40 °C (PDB ID: 5IZ7) (78). The surface structure has glycosylation at Asn154 on the E protein. The E protein arrangement exhibits a characteristic herringbone pattern of the flavivirus surface with 90 envelope-membrane protein (E-M)_2_ dimeric heterodimers (83, 84), including one (E-M)_2_ dimeric heterodimer positioned on each of the 30 vertices and 60 (E-M)_2_ dimeric heterodimers generally positioned within the icosahedral symmetry (79).

The cryo-EM structure of an immature ZIKV was reported in 2017 with 9 Å resolution (PDB ID: 5U4W), and the map showed the expected (85) spiky surface appearance resulting from the formation of 60 trimeric heterodimer “spikes” of the E and precursor M proteins (E-prM) (86). This trimeric form is held together at its tip *via* interaction between the pr domain of prM and the fusion loop of E proteins. The base of the prM-E spike is stabilized by the interaction between the amino acid residue of the EIII domain of E protein in one spike and EII domain of E protein from an adjacent spike (86).

### ZIKV-host interactions

Since the identification of the ZIKV in 1947 and the isolation of the MR766 prototype strain, many additional strains have been identified with sequence and structural changes (87, 88). Some of these changes may have contributed to the evolution of increased virulence in the ZIKV strains that have caused a series of more recent epidemics in many territories and countries (2007-2016) (88). The various modes of ZIKV transmission so far reported indicate that the virus has broad tissue tropism, and reports confirming the presence of infectious ZIKV particles in various human tissue and body fluids (89) provide supporting evidence of that. ZIKV enters the multiple host cell types *via* initial interaction of its envelope glycoprotein with various cell surface receptors shown to facilitate ZIKV entry (90). These receptors include AXL, TYRO3, DC-SIGN, and TIM1, where AXL is found to be the key receptor that plays the most significant role (91). The TAM ligand Gas6 acts as a cofactor to recruit the ZIKV particle to the ALX receptor, and then it is internalized by clathrin-mediated endocytosis (68). Afterward, the endocytic vesicles containing ZIKV move to the endosomes where transcription of several cellular genes is induced, such as DDX58, IFIH1, TLR3, and IFN stimulating genes. This results in suppression of the immune response and enhanced infection (68, 69, 91).

### Genome and polyprotein

The ZIKV genome is ~10.7 kb positive-sense single-stranded RNA released into the cytoplasm of host cells after the E protein-mediated fusion of viral membrane to the host endosomal membrane. Then, translation of the RNA into a single polypeptide chain occurs at the ER. This polypeptide chain includes a sequence of all the viral proteins from the N to C terminus as C-prM-E-NS1-NS2A-NS2B-NS3-NS4A-NS4B-NS5. The polypeptide is further hydrolyzed by viral and host proteases into three structural and seven non-structural proteins. The structural proteins include the capsid (C), precursor membrane (prM), and envelope (E), while the non-structural proteins include NS1, NS2A, NS2B, NS3, NS4A, NS4B, and NS5 (92, 93).

#### Structural proteins

X-ray crystal structures of dimeric E proteins (PDB ID: 5LBV and 5JHM) revealed that each monomer consists of three domains DI, DII, and DIII, where DI is the central domain linking the DII to DIII (94, 95). Domain DII includes a fusion peptide with a conserved amino acid sequence that interacts with the host endosomal membrane during the fusion of virus particles. Additionally, many residues of the DII domain are also critical in forming hydrogen bonds and electrostatic interactions necessary for stabilizing the E protein dimers (94). Domain DIII is involved in the primary interaction of the virus with the host cells and includes the receptor binding site. Thus, DIII plays a vital role in the fusion of viruses during cell entry (96-98). In flaviviruses, the flexible DI-DII hinge allows the fusion loop to be exposed during fusion events. Previously it was believed that the DI-DIII hinge is rigid, and conformational changes occur with fusion of E protein in its trimeric state (97, 98). In 2018, an improved 3.1 Å resolution cryo-EM map of the mature ZIKV (PDB ID: 6CO8) showed that E protein has three β-barrel domains corresponding to DI, DII, and DIII, which are anchored in the membrane by two transmembrane helices *via* three stem helices (99). The ZIKV E DI domain consists of three helices and nine β-strands, glycosylation on Asn154. The DII domain has two helices and nine β-strands with a fusion loop on residues 98-109 that is hydrophobic and highly conserved among flaviviruses, and the DIII domain has only seven β-strands (99).

The membrane (M) protein is divided into regions MH1, MH2, and MH3, where MH2 and MH3 are transmembrane helices. A loop is formed by N-terminal residues of the M protein, which interacts with the DII domain of E protein (99). An ordered nucleocapsid core is not detected in ZIKV or other mature flavivirus cryo-EM maps (100, 101). However, the 9 Å resolution immature cryo-EM structure of ZIKV has revealed a partially ordered capsid protein shell (86). Furthermore, a more recent 8 Å resolution cryo-EM map of immature ZIKV (102) has shown that the capsid protein interacts with the transmembrane regions of M and E proteins. The crystal structure of the ZIKV capsid protein shows that it has a stable dimeric conformation with an extended loop at the N-terminus, with a few amino acid residues at N-terminal and C-terminal are unresolved in the structure (103). The capsid protein plays an important role in the maturation of the virus particles (104) and the packaging of the genomic RNA in the virus core (103). However, the functional mechanisms of RNA packaging and release are yet to be explored in great detail for ZIKV or other flaviviruses.

#### Non-structural proteins

NS1 and NS2A: The crystal structure of NS1 shows three domains, namely N-terminal β-roll, an epitope-rich wing, and a C-terminal β-ladder (105). The membrane-bound NS1 forms a homodimer which is stabilized by an interaction between β-ladder and β-roll and secreted as a hexamer. A hydrophobic core is formed by the β-roll and the greasy finger motif on the inner face of the homodimer. NS1 interacts with the membrane *via* this hydrophobic core. The outer face is polar, where residues at positions 130 and 207 have been identified as glycosylation sites (105, 106). NS1 is translocated into the ER lumen during translation, where the host cell signal peptidase hydrolyzes the NS1 N-terminus, and the junction between NS1 and NS2A is cleaved by an unknown host protein. Hydrolysis of the NS1-NS2A junction is believed to be a good target for inhibitor development (107). Additionally, the hydrophobic core of the NS1 is thought to be a good target due to its ability to mediate the interaction between NS1 and host cell lipids (108). NS1 is secreted as a lipoprotein hexamer from host cells and interacts with adaptive and innate immune system components. Thus, it may modulate the host immune response and viral pathogenesis (108).

NS1 is also involved in forming the replication complex at the ER together with NS4A and NS4B (108), and it interacts with the prM and E structural proteins (107). The NS2A protein plays a central role in viral RNA replication and virion assembly, where NS2A has been shown to orchestrate virion morphogenesis by recruiting viral RNA, structural protein prM and E, and the NS2B/NS3 protease to the virion assembly site (109).

NS2B and NS3: The ZIKV NS2B is a membrane protein (130 residues long) consisting of transmembrane domains and a cytosolic domain like other flaviviruses (110-113). The cytosolic domain is hydrophilic and acts as a co-factor for NS3 protease activity. In contrast, the transmembrane domain is hydrophobic and tightly associated with ER membrane, providing a membrane anchor for NS3 protease (113-115). It was recently reported that serial passaging of ZIKV in mosquito cells and mice produced the emergence of the ZIKV strain with an I39V point mutation in NS2B that conferred enhanced transmissibility and pathogenicity to the virus. Similarly, an I39T mutation has been detected in mosquito isolates, and both mutations at the 39^th^ position were shown to increase replication of ZIKV in human NPCs and mosquitos (116). Thus, NS2B could be an essential protein of interest in efforts to prevent the emergence of a more transmissible ZIKV variant in the future.

NS3 is a larger protein of 617 amino acids, where the N-terminal region from residues 1 to 170 has protease activity required for polyprotein hydrolysis (117), while the C-terminal region of this protein from residues 171 to 617 has helicase activity and NTPase activity (118). Given that NS3 is both a protease and a helicase, it is considered an excellent target for developing antiviral drugs due to its multifunctional role in virus replication (119, 120).

NS3 protease domain: NS3 protease activity is vital for the proteolytic processing of the single polyprotein encoded by the ssRNA genome (121-123). For functional activity, this enzyme requires NS2B as a co-factor, and together these two proteins form the NS2B-NS3 protease complex (117). NS2B recruits NS3 to the ER membranes and is essential for the folding and catalysis of the protease complex (124, 125). The NS2B-NS3 protease cleaves the polyprotein at six sites, including a peptide bond within the capsid and the five peptide bonds between NS2A/NS2B, NS2B/NS3, NS3/NS4A, and NS4B/NS5 (126). Many high-resolution crystal structures of the NS2B-NS3 protease have been solved to understand the structural properties (117, 125, 127). The NS2B-NS3 protease structure (PDB ID: 5LC0) includes the hydrophilic region of NS2B, with residues 49–95 fused *via* a Gly4–Ser–Gly4 linker to the NS3 protease N-terminus (117), which shows that NS2B wraps around the NS3 protease in such a way that the C-terminal residues of the hydrophilic region of NS2B form a β-hairpin lying near the S2 pocket of the NS3 protease (117, 128, 129).

**FIGURE 1:**
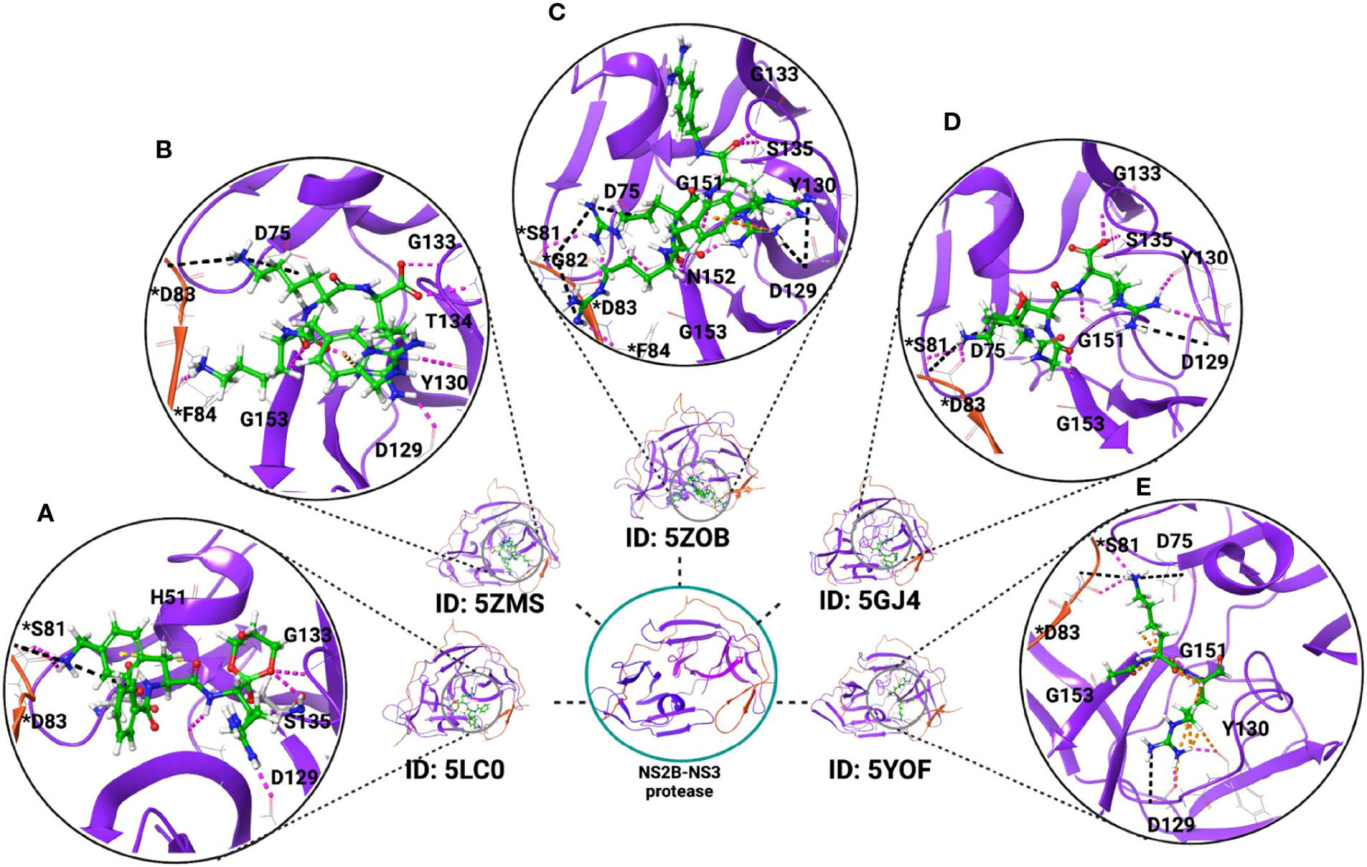
Crystal structure of ZIKV NS2B-NS3 protease in complex with inhibitors. These inhibitors reduce the protease activity and may block polyprotein cleavage in the host cells and could be potential inhibitors of ZIKV if tested in mammalian cells. Examples include the X-ray crystal structures of the NS2B-NS3 protease in complex with (**A**) the boronate inhibitor cn-716 (PDB ID: 5LC0); (**B**) 4-guanidinomethyl-phenylacetyl-Lys-Lys-Arg-H (PDB ID: 5ZMS); (**C**) 4-guanidinomethyl-phenylacetyl-Arg-Arg-Arg-4-amidinobenzylamide (PDB ID: 5ZOB); (**D**) Thr-Gly-Lys-Arg, tetrapeptide of NS2B C-terminal (PDB ID: 5GJ4); and (**E**) dipeptide inhibitor (PDB ID: 5YOF). Residues with an asterisk are from NS2B (red-colored ribbon), and residues without an asterisk are from NS3 protease (purple-colored ribbon). Inhibitors are represented in green color.

The NS2B-NS3 protease forms an unusual dimer with two-fold symmetry stabilized by a Cys143 disulfide bond on the NS3 protease. It also forms nine hydrogen bonds (Asp83, Ser81, Asp79, and Asp50 of NS2B; Asn158, Asp129, Thr27, and Leu30 of NS3 protease) at the dimerization interface and hydrophobic contacts (by Leu30 and Leu31 of NS3 protease) between opposing monomers (117). The NS3 protease active site possesses a catalytic triad of Ser135-His51-Asp75 conserved in other flavivirus members such as WNV and DENV. Additionally, the 83^rd^ residue from co-factor NS2B plays an essential role in protease activity as mutation at this position decreases the activity of the NS2B-NS3 protease compared to the wild type (117). Thus, the NS3 protease enzyme activity depends on the interaction of the substrate with the key amino residues from both the NS3 protease and NS2B. Depending on the binding of substrates, the NS2B-NS3 protease can adopt two conformations. In the presence of substrate, NS2B forms a β-hairpin that lies near the substrate-binding site of the NS3 protease and exists as a closed conformation, and in the absence of substrate, NS3 exists as an open conformation (117, 129-131). Besides the proteolytic processing of the single polyprotein, the NS2B-NS3 protease complex could play other functional roles, including involvement in the replication complex formation, interaction with other viral proteins NS4B and NS5, and in the modulation of host immune response and thus pathogenesis (117, 132-134). The NS2B-NS3 protease also suppresses the cGAS/STING signaling pathway and inactivates the antiviral defense. Due to its multiple roles in viral replication, the protease domain is considered an excellent target for identifying potential drug candidates (135-137). So far, many crystal structures of the NS2B-NS3 protease in complex with inhibitors and other compounds have been determined (117, 125, 132, 138-141) which will further help in guiding the development of ZIKV inhibitors. Figure 1 illustrates the interactions of a few compounds with the NS2B-NS3 protease, which are capable of inhibiting its protease activity. By inhibiting the activity of the NS2B-NS3 protease, these molecules may also inhibit the cleavage of the polyprotein into individual proteins and thus ZIKV replication. The potential of such inhibitors to work against ZIKV will require additional investigation in mammalian cells (117, 132, 141). In this regard, a few inhibitors have been identified that have the potential to inhibit NS2B-NS3 protease activity along with inhibition of ZIKV replication (142-145).

NS3 helicase domain: The NS3 helicase domain is responsible for unwinding the RNA structure during the viral RNA synthesis in coordination with NS5 polymerase. Meanwhile, the NTPase activity of the NS3 helicase provides energy to unwind RNA intermediates during replication (134, 146). Many high-resolution crystal structures have been determined for the NS3 helicase domain of flaviviruses to gain insight into its function (147-152). ZIKV NS3 helicase shows 70% primary sequence identity with DENV2 NS3, and it is observed to be a monomer in solution with three major regions: DI (residues 175-332), DII (residues 333-481), and DIII (residues 482-617) (152, 153). Crystal structure analysis reveals that residues 193-202 and 249-255 showed a high B-factor, suggesting higher flexibility in these regions. Additionally, the priming loop adopts varying conformational states among flaviviruses when not bound with ATP/Mg^2+^ (152, 153). An ATP/Mg^2+^ binding site is found in the cleft between the NS3 domain DI and DII, where residues G197, K200, T201, R202, E286, N330, R459, and R462 are involved in the binding interaction (152, 153). The binding site of ss-RNA is located in the tunnel from DII to DI and maintains a continual positive charge (152, 153). There are eight structural motifs on the ZIKV NS3 helicase domain. Their functions include involvement in ATP hydrolysis, communication between the binding sites, and RNA binding (134, 150, 152). NS3 helicase residues involved in the catalytic activity are conserved among flaviviruses, suggesting that the functions of this protein are preserved in the flavivirus (153). The structural characterization of the NS3 helicase suggests that the substrate-binding site, the RNA binding site, and ATP/Mg^2+^ binding site would be important targets for inhibitor design (152). Previously, some small molecules and nucleoside analogs have been identified that possess inhibition potential to the helicase activity of the HCV (154-156). Similarly, small molecules which contain benzothiazole and pyrrolone scaffolds have been reported to inhibit the NTPase and helicase activities of NS3 helicase in DENV (157).

NS4A and NS4B: The flavivirus NS4A protein plays a vital role in recruiting and assembling the replication complex to the ER by inducing membrane alteration of the host (158-160). NS4A is also believed to control the NS3 helicase activity (161, 162) and interacts with the NS1 protein to control viral replication (163). In ZIKV, NS4A and NS4B deregulates Akt-mTOR signaling pathways and induce autophagy to promote viral replication (164). The NS4B in Dengue virus interacts with the NS3 helicase to dissociate the helicase from ssRNA (165, 166). The ZIKV inhibitors, such as indole alkaloid derivatives, can target NS4B protein (167).

NS5: The largest protein encoded by the flavivirus genome is NS5. It has a crucial function in flavivirus replication and is thus a potential target for inhibitor development. Various crystal structures of NS5 are reported, showing two structural domains that perform a specific function in replication: the methyl transferase (MTase) domain in the N-terminus and the RNA-dependent RNA polymerase (RdRp) domain in the C-terminus. The MTase domain of NS5 methylates the mRNA cap at different positions, which enables the virus to evade detection by the host’s innate immune response system, and is thus essential for viral replication (168). This makes the MTase domain a promising target for inhibitors (169). Crystal structures of the ZIKV MTase domain have been determined in free form or complex with substrates and cofactors (170-173), showing that the structure consists of a Rossmann fold, as seen in other flaviviruses. Four conserved residues, K61, D146, K182, and E218, have been identified as a catalytic tetrad, which forms an active site on the ZIKV MTase domain of NS5 (174). Given that flavivirus MTase structures possess a high sequence identity with highly conserved residues in the binding site of S-adenosyl-L-methionine (SAM) and mRNA and nearly identical structures, this region is an excellent target for potential inhibitors that could be effective against all flaviviruses (173, 175).

The RdRp domain of NS5 carries out viral replication, and negative-sense RNAs are synthesized from the viral positive sense RNA template within the host cells. Subsequently, the negative-strand RNA is used as a template to make new copies of positive-sense RNA. The newly synthesized positive-strand RNAs are either used for translation or packing to form nascent virus particles. NS5 interacts with the viral NS3 protein and other host proteins (146, 178). It also acts as an antagonist for the IFN response (175-177). Diversity in the functional roles of NS5 makes the RdRp domain a key target for antiviral therapeutics against ZIKV and other flaviviruses. Several structures of the ZIKV NS5 RdRp domain have been reported, providing key information that can be used to compare ZIKV NS5 with that of other flaviviruses (175, 179, 180). The ZIKV RdRp adopts a right hand-shaped structure conserved structurally among flaviviruses. The RdRp of ZIKV consist of finger (321-488 & 542-608), thumb (715-903) and palm (489-541 & 609-714) subdomains (179). A catalytic site on the RdRp is located at the intersection between the finger domain and thumb domain. Two aspartic acids positioned at residues 535 and 665 are associated with the binding and positioning of the two zinc ions (Zn^2+^) in the catalytic site that catalyzes the nucleotidyl transfer (179), which has also been reported in other flaviviruses (181). These two ions make coordinate bonds with the residues of the finger subdomain (G439, H443, C448, and C451) and thumb subdomain (H714, C730, and C849) (179). Another important region is the priming loop (V785-D810) required for the allosteric placement of the RNA 3’-terminus into the active site (179). Additionally, it contains the nuclear localization signal (NLS) distributed between the thumb and finger subdomain, which mediates the transport of NS5 to the nucleus (175). Also, the NLS region is believed to facilitate interactions with the viral NS3 protein and host proteins (146, 178). Sequence comparison of the ZIKV NS5 RdRp with other flavivirus sequences has revealed that the priming loop located on the thumb subdomain, the Zn^2+^ pocket site, and the RNA binding tunnel located beneath the flexible loop of the finger subdomain, are among the most conserved regions (175). The RNA template entry tunnel and the N pocket located at the thumb subdomain near the active site of RdRp have been used as targets for inhibitors (175, 182-185).

## Cellular response to ZIKV infection with respect to drug targets

At the onset of viral infection, the innate immune system activates an acquired immunity response. To recognize the virus, the host innate immune system relies on pattern recognition receptors (PRRs) (186), such as retinoic acid-inducible gene I (RIG-I)-like receptors (RLRs), Toll-like receptors (TLRs), and NOD-like receptors (NLRs) (186, 187). These receptors recognize genomic and protein components that originate from an infecting virus (187, 188), which in turn triggers inflammation and the antiviral immune response. The recognition of these viral components by RLRs and TLRs induces the secretion of various cytokines, chemokines, and type I IFNs, while NLRs are involved in regulating interleukin-1b (IL-1b) maturation. Type I IFNs regulate the expression of various sets of genes by activating the intracellular signaling pathway (188, 189) whereas Type III IFNs (IFN-λ1, IFN-λ2, and IFN-λ3) are important in inducing antiviral responses, specifically during ZIKV infection (37). It has been reported that the primary human trophoblasts (PHT) cells act as a barrier to ZIKV infection. Simultaneously, the uninfected PHT cells also have a protective role for the non-placental cells. Findings suggest that the constitutive release of IFN-λ1 from the PHT cells protects both non-trophoblast and trophoblast cells against ZIKV infection (37). Further investigation in a mouse model and human epithelial cells from the cervix and vagina revealed that IFN-λ1 protects against ZIKV infection by inducing host defense transcriptional signature that controls infection, thus protecting the female reproductive tract and possibly reducing sexual transmission of ZIKV in women (190).

During abnormal congenital ZIKV syndrome (CZS), increased expression of IFIT5 occurs in the placenta and acts as an important enhancer for type I IFN (191, 193). A mouse model study demonstrated that dysregulation of the type I IFN response leads to CZS, suggesting that an optimal type I IFN response contributes to healthy pregnancy during ZIKV infection (38, 192). In addition, there is evidence that severe outcomes of CZS can be mitigated by the balanced production of host type I and type III IFN responses that protect against ZIKV infection in the placenta during pregnancy (193). While the host’s innate immune response has evolved several mechanisms to eliminate a virus, viruses are also constantly evolving with counter-mechanisms to evade and antagonize the host’s immune response (194). The functional role of flavivirus nonstructural proteins contributes to the manipulation of different host signaling pathways that antagonize the immune response, thus enabling the progression of the infection (195, 196). ZIKV non-structural proteins NS1, NS4A, and NS5 interfere with the induction of type I IFN by downregulating NF-_k_B and IRF3 signaling (41). More specifically, NS5 inhibits the type I IFN signaling by targeting the transcriptional activator, STAT2, resulting in its degradation in the proteasome (40, 41). NS5 activates the type II IFN signaling by inducing STAT1-STAT1 homodimer formation that promotes inflammation (197). NS5 also acts as a potent suppressor of Type III IFN signaling (197).

The ZIKV NS2B-NS3 complex inhibits type I IFN production by promoting Jak1 degradation downstream of JAK-STAT signaling (198). Additionally, it also acts as an inhibitor for the virus-induced apoptosis that may be helpful in the replication of the virus (198). The non-structural proteins NS4B and NS1 are also involved in the suppression of type I IFN production by blocking the oligomerization of TBK1 (198). ZIKV antagonizes type I IFN (189, 190) in dendric cells by an NS1-dependent CD303 signaling mechanism (199). The association of ZIKV with neurological disorders may be linked to the inhibition of RIG-I required to initiate the innate immune response of the host (200). The ZIKV NS2A and NS4A proteins play an important role as antagonists of IFN-β by suppressing IFN-β promoter activity through downregulation of RIG-I-like receptors and the downstream MDA5/RIG-I signaling pathway. ZIKV NS1 also reduces IFN-β production by downregulating MDA5 and active RIG-I (201). Additionally, NS4A and NS4B appear to be involved in the deregulation of the Akt-mTOR signaling pathways leading to the inhibition of neurogenesis and induction of autophagy in fetal neural stem cells (164). Thus, the deregulation of the Akt-mTOR pathways could be implicated in ZIKV-associated microcephaly of the fetus (202).

Neuronal progenitor cell (NPC) proliferation, differentiation, and organ size have been shown to be under control of the Hippo signaling pathway (203), leading to the hypothesis that the dysregulation of Hippo signaling pathway during ZIKV infection could have an adverse effect on the developing eye and brain. It has been shown that during ZIKV infection, the Hippo signaling pathway is involved in the regulation of immune response and the process of inflammation. Thus, this pathway could be another therapeutic target for controlling ocular and neuronal inflammation. There is also data indicating that ZIKV infection could initiate a cross-talk between AMP-activated protein kinase–Hippo–TBK1 pathways, thus regulating antiviral and energy stress responses in oculo-neuronal inflammation (204).

## Therapeutics

Vaccine development. Recent epidemics of ZIKV have created a demand for the development of effective therapeutics. In response, many ZIKV vaccine approaches have been in development, with several candidates now at different phases of clinical trials for safety and efficacy (205-208). Among several vaccine candidates, the inactivated vaccine ZIKAVAC and VRC 705, a DNA vaccine, are in phase 2 clinical trials. The current status of a few promising vaccine candidates is given in Table 1. However, the development of these candidates is facing challenges. The co-existence of other flaviviruses that share structural and genetic similarities with ZIKV can lead to a poor neutralizing effect and the possibility of antibody-dependent enhancement (ADE) of infection and disease severity, as there is some evidence of this for DENV (207). Additionally, the waning of the ZIKV epidemic in recent years has made it challenging to gather ongoing support for further clinical trials and evaluation of the effectiveness of the vaccine candidates (222). Considering the safety concern related to ADE, engineered vaccines (AdC7-M/E-MutB & AdC7-M/E-MutC) have been recently developed (223) by incorporating a few point mutations into the conserved fusion loop residues (98-109) of the ZIKV. The MutB vaccine construct possesses D98N, N103T, G106F, L107K & F108W mutations, and MutC vaccine construct possesses D98N, N103T, G106L, L107E & F108W mutations. Both these candidates induce protective immunity in the mice model and can nullify the effect of ADE. In addition, they are also able to provide fetal protection from the challenges that arise due to the vertical transmission of ZIKV infection in pregnant mice (223). Therefore, similar strategies could also apply to other vaccine development platforms such as DNA, RNA, subunit protein, or virus vector vaccine candidates (223).

The recent development of a maternal vaccine (live attenuated 3’UTR-Δ10-LAV) has been reported to prevent ZIKV-induced congenital syndrome of the fetus in pregnant mice with no adverse effect on pregnancy and fetal development (224). Despite the promising result, it may cause a subtle adverse effect on fetal development, and further investigation needs to be carried forward before the trials in humans. Still, it could be a basis for designing strategies to develop a maternal vaccine for pregnant women. In this context, the first DENV vaccine, ‘Dengvaxia,’ for treating secondary dengue infection (225) could be used as a standard for developing the ZIKV vaccine. Due to these challenges with vaccine development, parallel efforts to discover new inhibitor molecules that can function as therapeutics becomes more critical for the ZIKV-infected patient.

**FIGURE 2:**
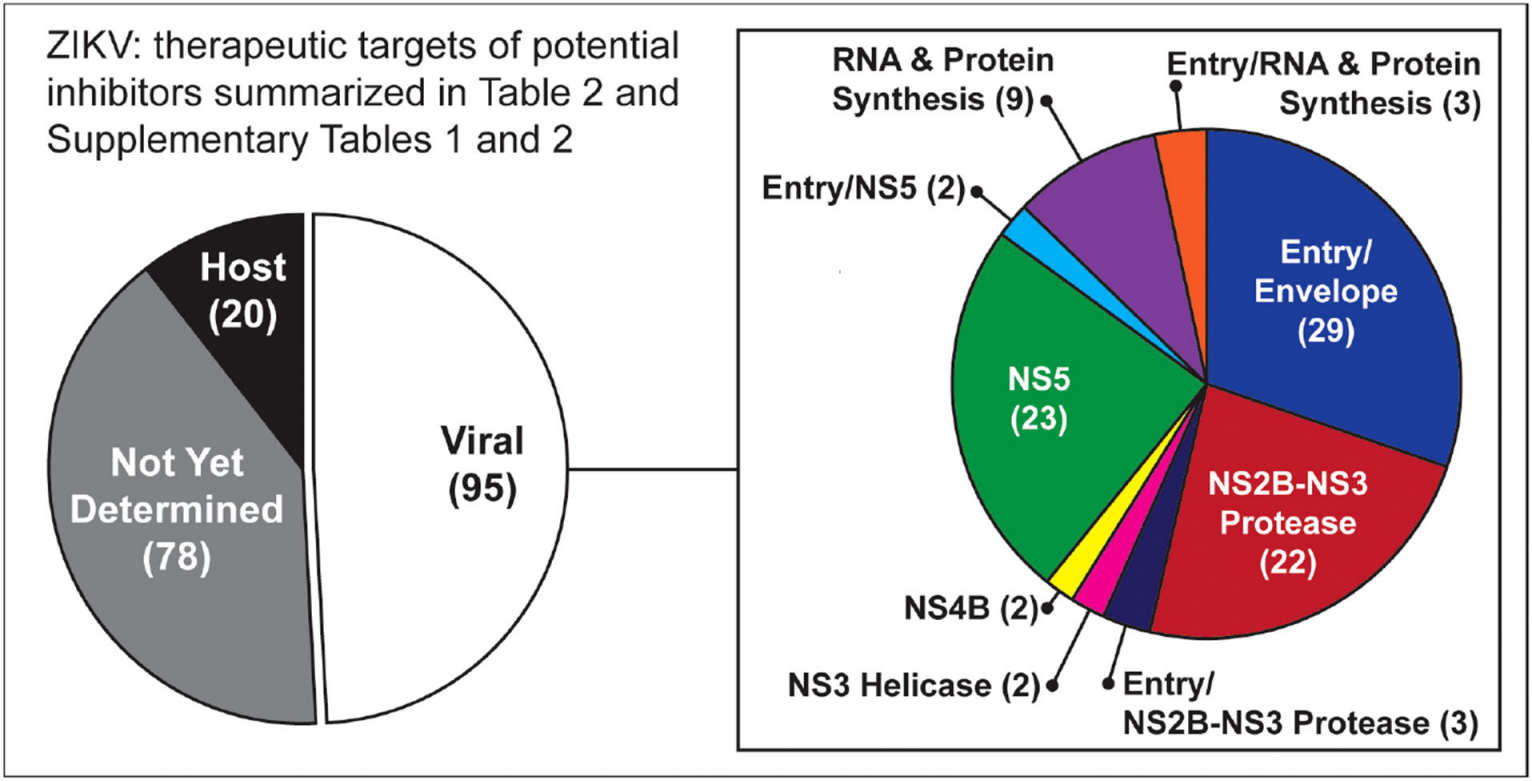
Graphic summary of targets for potential inhibitors listed in Table 2 and Supplementary Tables 1, 2. The most common viral protein targets for those inhibitors are NS2B-NS3 protease, NS5, and E or other entry-related proteins.

Drug screening: No novel drugs have been developed and specifically approved to treat ZIKV infection, so repurposing the existing drugs already approved for use in a diseased condition has been considered a useful, cost-effective, and expeditious path to finding effective therapeutics. In this review, we have summarized nearly 200 inhibitors, including many already approved by the U.S. Food and Drug Administration (FDA), that represent promising candidates for further investigation as repurposed therapeutic approaches to managing the pathogenesis of ZIKV infection. These compounds inhibit the ZIKV by targeting both the viral and host proteins/factor. The most common viral protein targets for those inhibitors are NS2B-NS3 protease, NS5, and E or other entry-related proteins (Figure 2). The list includes several inhibitors with activity that suggests they may prevent vertical transmission of the virus (Table 2).

**FIGURE 3:**
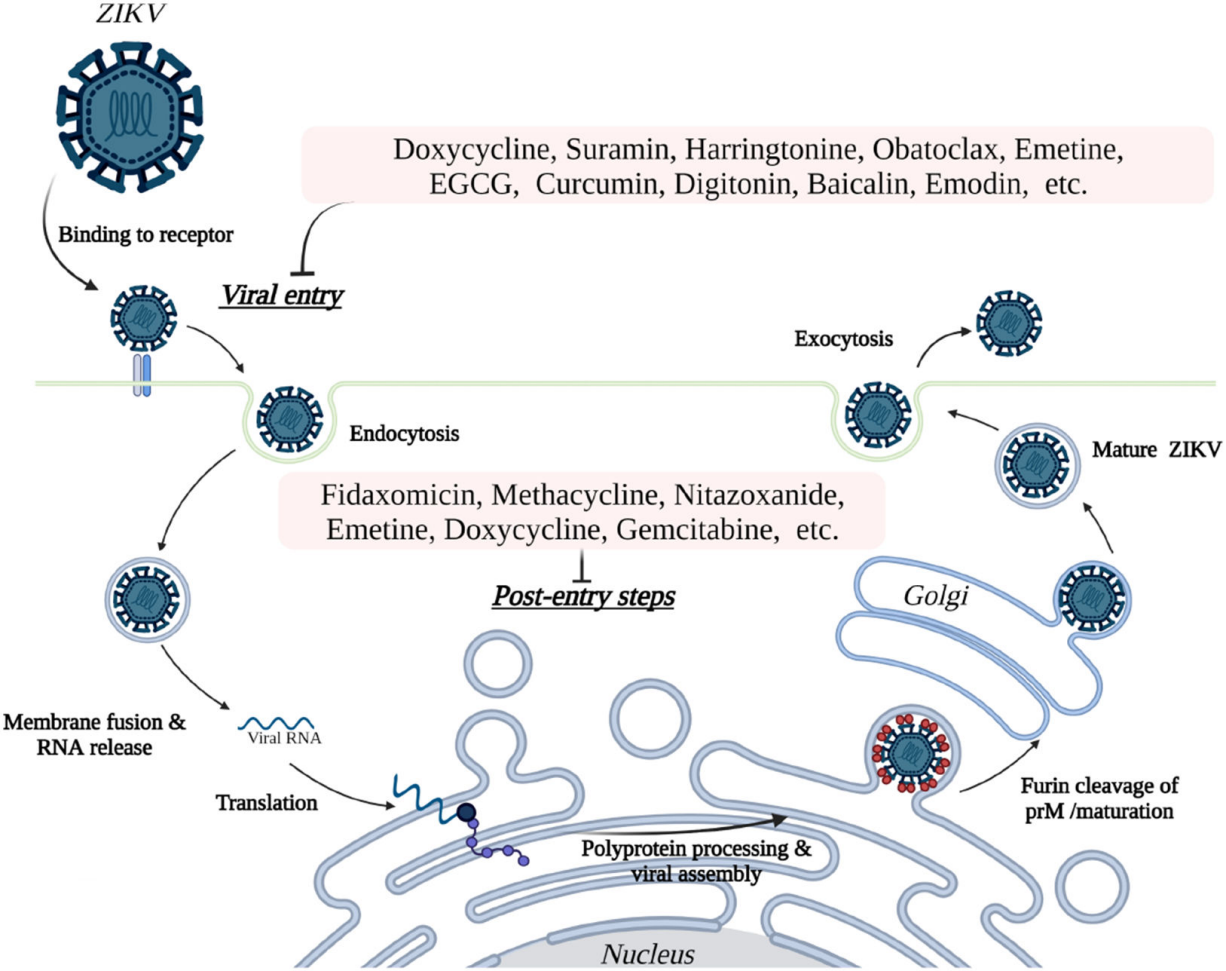
Inhibition of ZIKV entry and post entry steps into host cells. ZIKV initiates entry to the host cells by binding to cell surface receptors, including AXL, TYRO3, DC-SIGN, and TIM1. The subsequent endocytosis, fusion, and release of virus particles followed by replication of the RNA genome proceeds in the cytosol and endoplasmic reticulum (ER), respectively. Translation of the genome into a single polyprotein and processing of the polyprotein takes place on the ER membrane. Assembled viral particles bud into the ER lumen as immature viruses and proceed to maturity at the Golgi apparatus, then are released from the cell by exocytosis to perpetuate the cycle of infection. The potential inhibitors of ZIKV (see Table 2 and Supplementary Tables 1, 2) in mammalian cells inhibit the virus replication both at the host cell entry related steps and post-entry steps.

**FIGURE 4:**
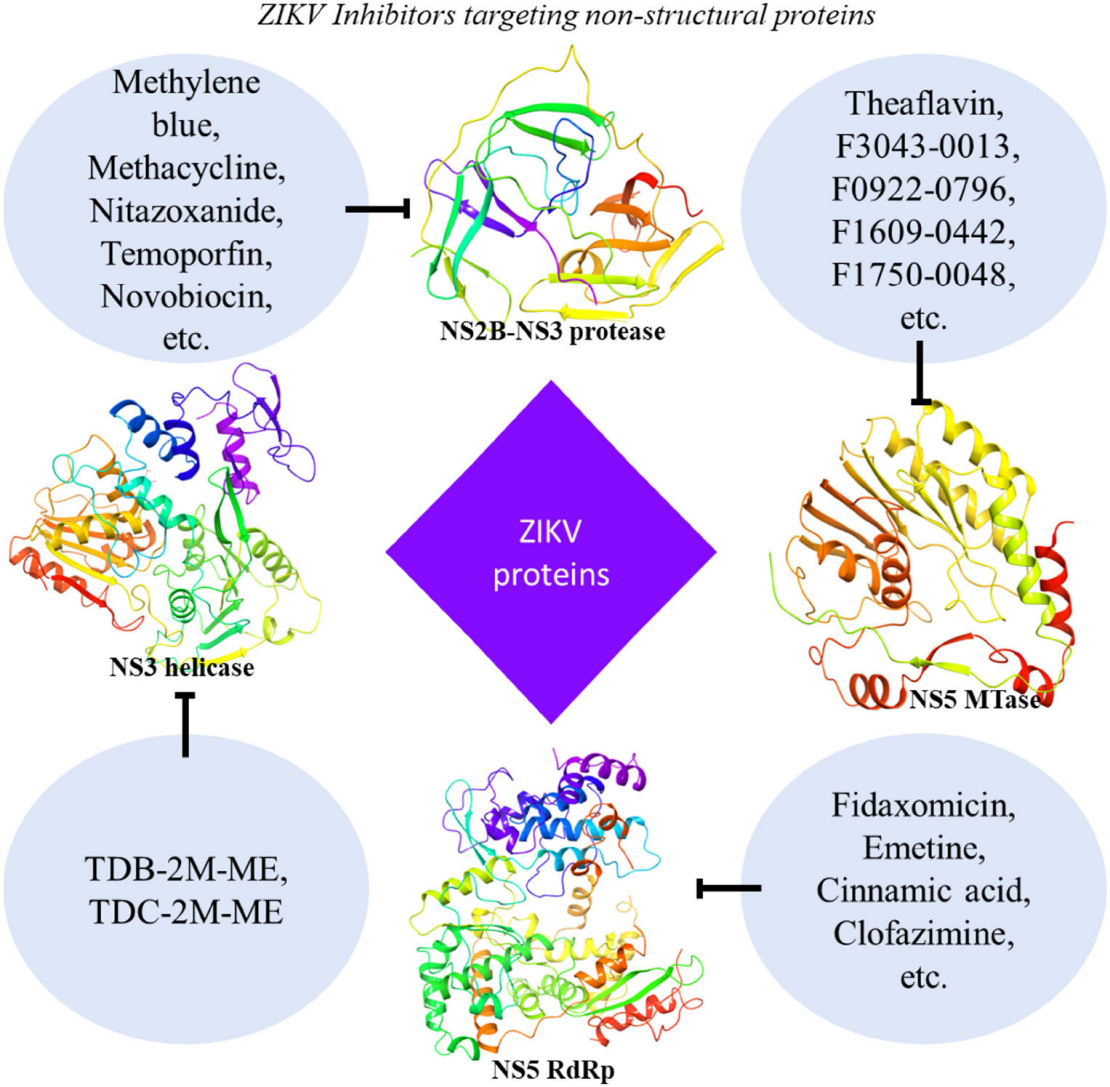
ZIKV Inhibitors targeting viral non-structural proteins. Inhibitor molecules, including FDA-approved drugs, natural products, nucleoside analogs, various derivatives, small molecules, and others, show the potential to inhibit ZIKV replication *in vitro* or *in vivo* (see Table 2 and Supplementary Tables 1, 2). These molecules target the non-structural (NS2B-NS3 protease, NS3 helicase, RdRp, and MTase) proteins of the ZIKV. Shown protein structures are based on PDB X-ray crystal structures extracted from Maestro software (NS2B-NS3 protease: 5H4I; NS3 helicase: 6MH3; RdRp: 5WZ3; MTase: 5WZ1).

Additional compounds with potentially beneficial properties include natural products, nucleoside analogs, peptides, small molecules, antibiotics, and other compounds that have been identified as possible inhibitors of ZIKV in both *in vivo* and *in vitro* studies (Supplementary Table 1) or only *in vitro* studies (Supplementary Table 2). Some of these inhibitors have demonstrated the ability to inhibit entry of the virus by targeting the envelope protein and entry related steps (Figure 3), while others inhibit the replication or assembly of the virus by targeting the non-structural proteins (Figure 4). The effectiveness and safety of these drugs and molecules deserves further evaluation in the pregnant mouse model to begin addressing questions about potential human use during pregnancy.

We present a brief overview of select drugs that show promise for preventing vertical transmission of ZIKV as well as inhibiting infection.

Chloroquine (CQ) is an FDA-approved antimalarial drug that has been repurposed to treat other conditions with some success (235, 236). In the COVID-19 pandemic, CQ and its analog hydroxychloroquine (HCQ) were initially reported to show an inhibitory effect *in vitro* on the SARS-CoV-2 replication (237, 238). But additional studies later suggested that the use of HCQ increases the mortality in the COVID-19 patient, whereas CQ does not exert any beneficial effect (239, 240). In contrast to the waning support related to the use of CQ or HCQ for COVID-19, the antiviral properties of these drugs in the case of ZIKV are more promising. Previously, CQ has demonstrated antiviral effects in various human cell types in cell culture, including trophoblast cells from the placenta (142), brain endothelial cells, neural stem cells, as well as in mouse neurosphere cells (56). In addition, experiments in fibroblast cells (BHK-21) show that CQ inhibits the entry and internalization of ZIKV (226). The administration of CQ to pregnant mice also significantly reduced ZIKV vertical transmission, with an observed 20-fold reduction in the virus load in the fetal brain (227). It has also been demonstrated that CQ can inhibit the early stage of ZIKV infection and provide protection from ZIKV-associated microcephaly in fetal mice (226). Overall, CQ shows inhibitory potential against ZIKV in reducing the viral load both *in vitro* (IC_50_ or EC_50_: 1.72-14.20 μM) and *in vivo* (56, 142, 226, 227), suggesting that this drug is a promising candidate for use as prophylaxis and in the treatment of patients with ZIKV infection (227).

Hydroxychloroquine (HCQ) is another FDA-approved antimalarial drug, similar to CQ, that has also been proven safe for pregnant women. This drug has been repurposed as an antirheumatic drug (241) and for treating DENV infections (242). Recent evidence suggests that HCQ inhibits the ZIKV infection in placental cells and limits the vertical transmission in pregnant mice by inhibiting the autophagy pathways (142). Additionally, a cell-based study suggests that HCQ reduces the burden of ZIKV in infected placental cells (80 μM significantly reduces Paraiba/2015 infection) due to the inhibition of the NS2B-NS3 protease activity (143).

Sofosbuvir (SOF) has been clinically approved for the treatment of hepatitis-C virus (HCV) (243). Because this is a class B FDA-approved drug safe for pregnant women and the fetus, several research groups have pursued it as a repurposed inhibitor drug candidate for ZIKV. So far, there is evidence that SOF targets the ZIKV RdRp (232) and inhibits replication of the virus in various cell systems (IC_50_ or EC_50_: 2.1-30.9 μM), including neural stem cells, human hepatoma cells, neuroblastoma, human liver cells, placental cells, and human brain organoids (229-232). Oral absorption or intraperitoneal administration of SOF was shown to reduce the death of the ZIKV-infected mice (228, 229). Further evidence suggests it plays a role in preventing short- and long-term behavioral changes sequelae in infected mice (228). Treatment with SOF also reduced the viral burden and increased the percentage and time of the survival of the infected animals (228, 231) while also preventing acute neuromotor abnormalities (228). A recent report also suggests its role in preventing ZIKV transmission from mother to fetus in pregnant mice (231).

Ouabain is an FDA-approved steroid hormone that has shown some potential as a ZIKV inhibitor candidate in a mouse model. Ouabain blocks the ZIKV infection (IC_50_: 48.39 nM for H/PF/2013) by targeting Na^+^/K^+^-ATPase at the replication stage and thus reduces the ZIKV load in adult mice and the placenta. Furthermore, it can penetrate the placental barrier and provide protection to fetal mice from ZIKV-induced microcephaly (233). The safety profile of this drug for pregnant women has not yet been demonstrated.

E protein peptide (Z2) has also demonstrated the ability to inhibit the ZIKV transmission from mother to fetus in mice. Z2 is a small peptide derived from the stem region of the ZIKV E protein (residues 421-453), and it interacts with the viral E protein resulting in disruption of the ZIKV membrane integrity. It is safe to use in pregnant mice and also inhibits the vertical transmission of ZIKV in C57BL/6 mice (234).

The following are other notable examples of drugs from Supplementary Table 1 that show promise for preventing ZIKV infection:

Methacycline is a type of tetracycline antibiotic that reportedly reduces ZIKV load in the brain and the severity of motor deficits in a mouse model (244). Additionally, it also inhibits the activity of ZIKV protease and reduces ZIKV infection (IC_50_: 7.3 μM for French Polynesian_2013) in NSCs.

Fidaxomicin is another antibiotic that is used clinically to treat infection of *Clostridium difficile* (245) and has shown inhibition potential against ZIKV both *in vitro* (EC_50_: 6-14.5 μM) and *in vivo* (246). It targets the ZIKV RdRp, which inhibits the RNA synthesis, and thus it reduces the ZIKV load in the brain and testes of infected mice (246).

Ribavirin is a drug used to treat Hepatitis C virus infection (247), which exerts its antiviral effect by inhibiting RdRp activity (248). It also inhibits ZIKV replication in mammalian cells (10-80 μg/ml inhibits MR766 growth) and prevents ZIKV-induced death and apoptosis in cell culture. Ribavirin was also shown to abrogate the viral load in blood samples from STAT-1-deficient mice infected with ZIKV (249).

Emetine has been used as a potent amoebicide agent (250) and has also shown the ability to act as an antiviral candidate against many viruses (251, 252). Emetine has demonstrated the ability to inhibit ZIKV infection both *in vitro* (IC_50_: 8.74-52.9 nM) and *in vivo* (253). It inhibits NS5 polymerase activity of ZIKV and accumulates in the lysosome, where it disrupts lysosomal function and leads to the inhibition of viral entry (253).

Memantine is used for the treatment of Alzheimer’s disease in which it acts as an NMDR receptor antagonist (254). It has been shown to prevent ZIKV-induced neuronal cell death (30 μM reduces cell death to ~20%) without interfering with ZIKV replication in these cells. It can reduce the neurodegeneration and microgliosis in a ZIKV-infected mice brain and can prevent the increased intraocular pressure as a result of ZIKV infection. As a neuroprotectant, memantine could be a potent candidate for treating patients at high risk of ZIKV-induced-neurodegeneration (255).

Novobiocin is an antibiotic (256) in the coumermycin family that is known to inhibit bacterial DNA gyrase (257). The repurposing of this antibiotic has shown anti-ZIKV activity in Vero (IC_50_: 42.63 μM for PRVABC59) and Huh cells (IC_50_: 62.24 μM for PRVABC59), and increases the survival rate of ZIKV-infected mice. It shows stable binding with the NS2B-NS3 protease and thus may have the potential to act as an inhibitor of this protease in ZIKV (258).

Additional FDA-approved drugs such as hippeastrine hydrobromide (HH) (IC50: 3.62 μM) (259), amodiaquine dihydrochloride dihydrate (AQ) (IC50: 2.81 μM) (259), methylene blue (MB) (1.67-15 μM) (145), and temoporfin (0.01-3 μM) (144) have been shown to inhibit ZIKV in human neuronal progenitor cells (hNPCs), and also suppress virus replication in mouse models (Supplementary Table 1). MB also stalls NS2B-NS3 protease activity by inhibiting the interaction between NS3 protease and co-factor NS2B. Both HH and AQ are shown to inhibit the ZIKV in fetal-like forebrain organoids and mice brains (259), where HH inhibits ZIKV replication in the brain. MB and temoporfin target the NS2B-NS3 protease and inhibit the protease activity, and are shown to inhibit ZIKV at the entry and post-infection stage (144, 145).

### Concluding remarks and future directions

Since the first ZIKV outbreak in 2007, no newly developed therapeutics or vaccines have become broadly available specifically for the treatment or prevention of ZIKV infection. This has contributed to significant concerns, particularly among pregnant women, as the association of ZIKV with neurological abnormalities in the fetus has now been established after being first reported in 2015 during the Brazil outbreak. In addition to a surge of microcephaly cases, Guillain-Barre syndrome cases increased twenty-fold at this time, sparking accelerated drug discovery research in search of specific therapeutic regimes for ZIKV infection. To expedite the ZIKV drug discovery process, researchers have sought to repurpose inhibitors and other drugs previously developed and approved for other uses. Many such inhibitor candidates have shown the ability to target ZIKV proteins, including the envelope, NS2B-NS3 protease, NS3 helicase, NS5 MTase, and NS5 RdRp. A detailed analysis of the structural features revealed by several X-ray crystal structures of ZIKV proteins has enabled ongoing structure-based targeting efforts to identify other inhibitors. Many of these prospective therapeutics have also shown inhibition of ZIKV in various cell systems or mouse models. Additionally, a few drugs, such as Chloroquine, Hydroxychloroquine, Sofosbuvir, and Ouabain which are FDA-approved for other uses, have also shown some ability to inhibit ZIKV transmission from mother to fetus, or reduce the condition of microcephaly in the mouse model. Building on these results and given the large number of drugs with potential that we have been able to identify for summary here, there is rationale for further investigation of these drugs so that they can be more fully screened, evaluated, considered for clinical testing in human trials, and eventually advanced through the discovery pipeline. So far, few vaccine candidates have advanced beyond the early phases of clinical trials, and questions about demand and efficacy remain due to the waning epidemic and the antibody-dependent enhancement of the disease severity. These vaccine candidates will also face the added challenge of being clinically evaluated for their safety and efficacy in pregnant women. Although ZIKV cases have subsided since 2016, vulnerable populations are still affected by the persistence of the virus in some regions and the emergence of outbreaks in new regions. To meet current demands for treatment and prepare for the ever-present threat of future large-scale ZIKV epidemics should more transmissible variants emerge, research efforts must be sustained in drug discovery along with vaccine development so that improved strategies will become available for treating and preventing ZIKV infections.

## Supplementary Material

Supplementary Table 1

Supplementary Table 2

## Figures and Tables

**TABLE 1:** A summary of the ZIKV vaccine candidates under different phases of human clinical trials.

Vaccine candidate	Developer/sponsor	Clinical trial status	Type of vaccine	Ref.
ZIKAVAC	Bharat Biotech	Phase 2	Inactivated vaccine	(209)
ZPIV	Walter Reed Army Institute of Research	Phase 1	Inactivated vaccine	(210, 211)
VLA1601	Valneva Austria GmbH	Phase 1	Inactivated vaccine	(212)
rZIKV/D4Δ30-713	NIAID’s Laboratory of Viral Diseases	Phase 1	Live attenuated vaccine	(213)
VRC 705	NIAID’s Vaccine Research Center	Phase 2	DNA vaccine	(214)
VRC 319	NIAID’s Vaccine Research Center	Phase 1	DNA vaccine	(215)
GLS 5700	GeneOne life science;Inovio Pharmaceuticals	Phase 1	DNA vaccine	(216, 217)
mRNA 1325	Moderna Therapeutics	Phase 1/2	mRNA vaccine	(218)
ChAdOx1 Zika	University of Oxford	Phase 1	Live adenovirus recombinant	(219)
MV-ZIKA-RSP	Themis Bioscience	Phase 1	Live measles recombinant	(220)
AGS-v	SEEK, hVIVO, and NIH Clinical Center	Phase 1	Synthetic proteins vaccine	(221)

**TABLE 2:** ZIKV inhibitors that have the potential to inhibit vertical transmission from mother to fetus in a mouse model.

Compound	Cell line (anti-ZIKV activity of compound, ZIKV strain)	*In vivo* model (dose of compound; ZIKV strain)	Mechanistic insight	Description of compound	Ref.
Chloroquine (CQ)	hfNPCs (90 % inhibition at 6 μM, ZIKV^BR^), BHK-21 (10 μM, GZ01/2016), Huh-7 (IC_50_: 1.72 μM, GZ01/2016; IC_50_: 2.72 μM, FSS13025), Vero (IC_50_: 4.15 μM, GZ01/2016; EC_50_: 9.82 μM, MR766; at 25 μM, 16-fold reduction in ZIKV^BR^ RNA), hBMEC (EC_50_:14.20 μM, MR766), hNSC (EC_50_: 12.36 μM, MR766), Neurosphere (12.5 μM, decreases MR766 infection)	AG129 (50 mg/kg/day; ZIKV ^BR^), SJL (30 mg/kg/day; ZIKV ^BR^), BALB/c (100 mg/kg; GZ01/2016), A129 (100 mg/kg; GZ01/2016)	Inhibit viral entry to host cell.	FDA approved drug	(56, 226, 227)
Hydroxychloroquine (HCQ)	JEG3 (80 μM, reduces Paraiba/2015 infection)	C57BL/6 (40 mg/kg/day; Paraiba/2015)	Inhibitor of NS2B-NS3 protease (*in vitro* & *in silico*).	FDA approved drug	(142, 143)
Sofosbuvir (SOF)	hNPCs (IC50 : 13.6 μM, IbH-30656), Vero (IC50 : 30.9 μM, IbH-30656), Huh-7 (IC_50_ : 3.9μM, H/PF/2013; IC_50_ : 4 μM, ZIKVNL00013; EC_50_: 1.37 μM, PRVABC59; EC_50_: 3.8 μM, Paraiba/2015; EC_50_: 4.6 μM, Senegal 1984 ), Jar (EC_50_: 4.95 μM, PRVABC59; EC_50_: 2.1 μM, Paraiba/2015; EC_50_: 3.79 μM, Senegal 1984), NSCs (EC_50_ : 32 μM, Praiba), SH-Sy5y (EC_50_:1.1 U/ml; Brazilian ZIKV), BHK-21 (EC_50_:1.9 U/ml; Brazilian ZIKV), brain organoids	NOD/SCID (50 mg/kg/day; IbH-30656), SJL mice (50 mg/kg/day; Asian ZIKV strain 259459), Swiss albino mice (20 mg/kg/day; MR766), C57BL/6 (30 mg/kg/day; ZIKV Senegal 1984)	Inhibitor of RdRp (*in vitro* & *in silico*).	FDA approved drug	(228-232)
Ouabain	Vero (IC50: 48.39 nM, H/PF/2013; 49-784 nM, inhibit MRS infection), Huh-7 (24.5-784 nM, inhibit H/PF/2013 infection; 12.25-784 nM, inhibit MRS infection), U251 (24.5-784 nM, inhibit H/PF/2013 infection; 12.25-784 nM, inhibit MRS infection)	*Ifnar1*^−/−^ (2 mg/kg; H/PF/2013), C57BL/6 (3 mg/kg/day; H/PF/2013)	Targets Na^+^/K^+^-ATPase.	FDA approved drug	(233)
E Protein Peptide Z2	BHK-21 (IC_50_: 1.75 μM, SZ01; IC_50_: 4.04 μM, FLR; IC_50_: 13.91 μM, MR766) and Vero (IC_50_: 3.69 μM, SZ01)	A129 or AG6 (10 mg/kg; GZ01), C57BL/6 (10 mg/kg; SZ01)	Interacts with E protein and disrupts virus membrane (*in vitro*).	Peptide (stem region of ZIKV E protein)	(234)

[EC_50_= half maximal effective concentration; IC_50_= half maximal inhibitory concentration].
