# Supplementary material for: Drugs to limit Zika virus infection and implication for maternal-fetal health
Source: Front Virol. Author manuscript; Available in PMC 2023 Apr 14. (PMC10104533; doi:10.3389/fviro.2022.928599)
Supplement: Supplementary Table 1 [file NIHMS1839512-supplement-Supplementary_Table_1.docx]

**Supplementary Table 1.** A summary of compounds tested *in vitro* and *in vivo* with inhibition potential for ZIKV. [N.D. = not determined; EC_50_= half maximal effective concentration; IC_50_= half maximal inhibitory concentration]

| **Compound** | **Cell line (anti-ZIKV activity of compound, ZIKV strain)** | ***in vivo* model (dose of compound; ZIKV strain)** | **Mechanistic insight** | **Description of compound** | **Ref.** |
| --- | --- | --- | --- | --- | --- |
| Amodiaquine dihydrochloride dihydrate (AQ) | hNPCs (IC_50_: 2.81 µM, MR766), hPSC-derived forebrain organoids (15 µM, suppresses MR766 infection) | SCID-Beige mice (40 mg/kg/day; MR766) | N.D. | FDA approved drug | (1) |
| Hippeastrine hydrobromide (HH) | hNPCs (IC_50_: 3.62 µM, MR766), hPSC-derived forebrain organoids (25 µM, suppresses MR766 infection) | SCID-Beige mice (100 and 200 mg/kg/day for MR766; 100 mg/kg/day for FSS13025). | N.D. | FDA approved drug | (1) |
| Methylene blue | A549 (EC_50_: 0.2 µM, MR766; EC_50_: 0.87 µM, PRVABC59; EC_50_: 0.14 µM, FSS13025; EC_50_: 0.087 µM, MeX I-44; EC_50_: 0.14 µM, BeH819015), 3D mini-brain organoids (1.5 µM completely protected from PRVABC59) | IfnαβR−/− mice (100 mg/kg/day; PRVABC59) | Inhibit viral entry to host cell. Disrupts the interaction between NS2B and NS3 protease. Inhibitor of NS2B-NS3 protease (*in vitro*). | FDA approved drug | (2) |
| Temoporfin | A549 (EC_50_: 0.024 µM, PRVABC59), Vero (EC_50_: 0.1 µg/ml, GZ01/2016), HPECs (0.01-3 µM, PRVABC59), hNPCs (0.01-3 µM, PRVABC59), h-iPSC line HDF9 (0.04-3 µM, PRVABC59) | Balb/C mice (0.02 mg/mice/day; GZ01/2016), A129 mice (1mg/kg/day; GZ01/2016) | Disrupts the interaction between NS2B and NS3 protease. Inhibitor of NS2B-NS3 protease (*in vitro* & *in silico*). | FDA approved drug | (3) |
| Methacycline | NSCs (IC_50_: 7.3 µM, French Polynesian_2013) | C57BL/6 mice (10 mg/kg/day; Brazil_2015) | Inhibitor of NS2B-NS3 protease (*in vitro* & *in silico*). Reduces the severity of motor deficits. | FDA approved drug | (4) |
| Fidaxomicin | SNB19 (EC_50_: 6 µM, ZG-01; EC_50_: 6.8 µM MR766), A172 (EC_50_: 8.8 µM ZG-01), Vero (EC_50_: 11.7 µM, ZG-01; EC_50_: 12.4 µM MR766), Huh7 (EC_50_: 7.7 µM, ZG-01; EC_50_: 7.3 µM MR766), A549 (EC_50_: 12.2 µM, ZG-01; EC_50_: 13 µM MR766), HUVECs (EC_50_: 14.5 µM, ZG-01) | Ifnar1−/− C57BL/6 mice (10-20 mg/kg; ZG-01) | Inhibitor of RdRp (*in vitro* & *in silico*). | FDA approved drug | (5) |
| Ribavirin | Vero (80 µg/ml could inhibit MR766 replication almost completely), SH-SY5Y (10 µg/ml inhibit MR766 replication strongly), C6/36 (10 µg/ml inhibit MR766 replication significantly) | STAT1-deficient mice (15 mg/day; MR766) | N.D. | FDA approved drug | (6) |
| Emetine | SNB-19 (IC_50_: 29.8 nM, PRVABC59), HEK293 (IC_50_: 52.9 nM, MR766), Vero (IC_50_: 8.74 nM, PRVABC59) | SJL mice (1 mg/kg/day; ZIKV^BR^), Ifnar1−/− mice (2 mg/kg/day; FSS13025) | Disrupts lysosomal function to prevent virus entry. Inhibitor of RdRp (*in vitro* & *in silico*). | FDA approved drug | (7) |
| Memantine | Neuronal cells (30 µM reduces cell death to ~20 %, HS-2015-BA-01) | IFN-α/βR−/− mice (30 mg/kg twice daily; HS-2015-BA-01) | Blocks N-methyl-D-aspartate receptor (NMDAR). | FDA approved drug | (8) |
| Novobiocin | Vero (IC_50_: 42.63 µM, PRVABC59), Huh-7 (IC_50_: 62.24 µM, PRVABC59) | BALB/c mice (100 mg/kg q12h; PRVABC59) | Inhibitor of NS2B-NS3 protease (*in vitro* & *in silico*). | FDA approved drugs | (9) |
| TPB | [Vero](https://www.sciencedirect.com/topics/medicine-and-dentistry/embryonal-carcinoma) (IC_50_: 94 nM, PRVABC59; 0.5-2 µM, inhibits MR766 growth), HTR-8 (0.5-1 µM, inhibits PRVABC59 growth), NTERRA-2 (0.5-1 µM, inhibits PRVABC59 growth) | BALB/c mice (25 mg/kg; PRVABC59) | Interacts with RdRp (*in silico*). | Non-nucleoside inhibitor | (10) |
| BCX4430 | Vero (EC_50_: 11.7 or 8.7 µg/ml, MR766; EC_50_: 11.5 or 13.8 µg/ml, P 6-740; EC_50_: 3.8 or 18.2 µg/ml, PRVABC59), Huh-7 (EC_50_: 5.7 or 6.4 µg/ml, MR766; EC_50_: 5.5 or 4.9 µg/ml, P 6-740; EC_50_: 4.7 or 6.7 µg/ml, PRVABC59), RD (EC_50_: 4.4 or 5.4 µg/ml, MR766; EC_50_: 4.7 or 10 µg/ml, PRVABC59) | AG129 mice (twice daily at 300 mg/kg/day; P 6-740) | N.D. | Nucleoside analog | (11) |
| NITD008 | Vero (EC_50_: 241 nM, GZ01/2016; EC_50_: 137 nM, FSS13025; EC_50_: 0.50 µM, MR766; EC_50_: 0.56 µM PRVABC59) | A129 mice (50 mg/kg; GZ01/2016) | N.D. | Nucleoside analog | (12, 13) |
| 7DMA (7-deaza-2’-C-methyladenosine) | Vero (EC_50_: 1.3-20 µM, MR766) | AG129 mice (50 mg/kg/day; MR766) | N.D. | Nucleoside analog (viral polymerase inhibitor) | (14, 15) |
| N-ter-WDFGSIGGVFNSIGKAVHQVF-C-ter | Vero (IC_50_: 3.27 µM, H/PF/2013; IC_50_: 0.07 µM, MRS), Huh-7 (IC_50_: 0.55 µM, H/PF/2013; IC_50_: 0.13 µM, MRS) | C57BL/6 mice (50 µM; H/PF/2013) | N.D. | Peptide (derived from JEV E protein stem region) | (16) |
| AH-D peptide (alpha helix and D enantiomer) | Neuronal cells from C57BL/6J (IC_50_: 0.0119 µM; MR766, HS-2015-BA-01) | Ifn-α/βR−/− SV129 (25 mg/kg twice daily; HS-2015-BA-01) | Penetrates blood brain barrier to prevent brain damage and neurodegeneration. | Peptide | (17) |
| NSC157058 | hfNPCs (IC_50_: ~50 µM, FSS13025) | [SJL mice](https://www.sciencedirect.com/topics/pharmacology-toxicology-and-pharmaceutical-science/swiss-james-lambert-mouse) (30 mg/kg/day; PA259459) | Inhibitor of NS2B-NS3 protease (*in vitro* & *in silico*). | Small molecule | (18) |
| 25HC (25-hydroxycholesterol) | Vero (IC_50_: 188 nm, GZ01/2016), human cortical organoids (2.5 µM reduces 90% ZIKV RNA of PRVABC59) | BALB/c (50 mg/kg; GZ01/2016), A129 (50 mg/kg; GZ01/2016); Rhesus monkeys _Macaca mulatta (1.5 mg/kg/day; GZ01/2016) | Blocks viral entry to host cell and protect from microcephaly. | Enzymatic product of cholesterol-25-hydroxylase | (19) |
| Cinnamic acid | Vero (EC_50_: 49.55 µM, Asian_KU963796), Huh-7 (EC_50_: 83.96 µM, Asian_KU963796), A549 (EC_50_: 103.8 µM, Asian_KU963796) | AG6 (75 or 150 mg/kg; Asian_KU963796) | Inhibitor of RdRp (*in vitro* & *in silico*). | Organic acid | (20) |
| Methyl 5-chloro-3-(4-hydroxy-3,5-dimethoxybenzoyl)-1H-indole-2-carboxylate | Huh-7 (EC_50_: 13.9 µM, 976 Uganda) | ICR suckling mouse (1 mg/kg at 1,3 and 5 dpi; 976 Uganda) | Inhibitor (allosteric) of NS2B-NS3 protease (*in vitro* & *in silico*). Inhibits RNA synthesis and protein production. Inhibits brain damage. | Small molecule | (21) |
| JMX0207 | A549 (EC_50_: 0.30 µM, PRVABC59), hNPCs (7.5 µM, protects from PRVABC59 infection), cortical organoids (1.5 µM, completely protected from PRVABC59 infection) | A129 (20 mg/kg/day; PRVABC59) | Inhibit the molecular interaction between NS3 and NS2B. Inhibitor of NS2B-NS3 protease (*in vitro*). | Niclosamide derivative | (22) |
| Erythrosin B | A549 (EC_50_: 0.62 µM, PRVABC59), HPEC (EC_50_: 0.60 µM, PRVABC59), HPNC (EC_50_: 1.39 µM, PRVABC59), forebrain organoid (3 µmol/L, significantly reduces ZIKV infection, ZIKV-Venus) | A129 (200 mg/kg/day; PRVABC59) | Disrupts the interaction between NS2B and NS3 protease. Inhibitor of NS2B-NS3 protease (*in vitro* & *in silico*). | FDA approved food additive | (23, 24) |
| Cephaeline | HEK 293 (IC_50_: 26.4 nM, MR766), SNB-19 (IC_50_: 3.11 nM), astrocytoma cells (IC_50_: 7.60 nM, MR766; IC_50_: 10.51 nM, PRVABC59) | Ifnar1-/- (2mg/kg/day; FS13025) | Inhibitor of NS5 RdRp | Emetine analog | (7) |
| 6-bromo-1,2-naphthalenedione | Vero (50 µM, 150-fold reduction in MR766) | AG129 (50 µg/day; MR766) | Inhibitor of NS2B-NS3 protease (*in vitro*) | Small molecule | (25) |

Reference:

1. Zhou, T., Tan, L., Cederquist, G. Y., Fan, Y., Hartley, B. J., Mukherjee, S., Tomishima, M., Brennand, K. J., Zhang, Q., Schwartz, R. E., Evans, T., Studer, L., and Chen, S. (2017) High-Content Screening in hPSC-Neural Progenitors Identifies Drug Candidates that Inhibit Zika Virus Infection in Fetal-like Organoids and Adult Brain. *Cell Stem Cell*. **21**, 274-283.e5

2. Li, Z., Lang, Y., Sakamuru, S., Samrat, S., Trudeau, N., Kuo, L., Rugenstein, N., Tharappel, A., D’Brant, L., Koetzner, C. A., Hu, S., Zhang, J., Huang, R., Kramer, L. D., Butler, D., Xia, M., and Li, H. (2020) Methylene blue is a potent and broad-spectrum inhibitor against Zika virus in vitro and in vivo. *https://doi.org/10.1080/22221751.2020.1838954*. **9**, 2404–2416

3. Li, Z., Brecher, M., Deng, Y. Q., Zhang, J., Sakamuru, S., Liu, B., Huang, R., Koetzner, C. A., Allen, C. A., Jones, S. A., Chen, H., Zhang, N. N., Tian, M., Gao, F., Lin, Q., Banavali, N., Zhou, J., Boles, N., Xia, M., Kramer, L. D., Qin, C. F., and Li, H. (2017) Existing drugs as broad-spectrum and potent inhibitors for Zika virus by targeting NS2B-NS3 interaction. *Cell Res. 2017 278*. **27**, 1046–1064

4. Abrams, R. P. M., Yasgar, A., Teramoto, T., Lee, M. H., Dorjsuren, D., Eastman, R. T., Malik, N., Zakharov, A. V., Li, W., Bachani, M., Brimacombe, K., Steiner, J. P., Hall, M. D., Balasubramanian, A., Jadhav, A., Padmanabhan, R., Simeonov, A., and Nath, A. (2020) Therapeutic candidates for the Zika virus identified by a high-throughput screen for Zika protease inhibitors. *Proc. Natl. Acad. Sci. U. S. A.* **117**, 31365–31375

5. Yuan, J., Yu, J., Huang, Y., He, Z., Luo, J., Wu, Y., Zheng, Y., Wu, J., Zhu, X., Wang, H., and Li, M. (2020) Antibiotic fidaxomicin is an RdRp inhibitor as a potential new therapeutic agent against Zika virus. *BMC Med.* **18**, 1–16

6. Kamiyama, N., Soma, R., Hidano, S., Watanabe, K., Umekita, H., Fukuda, C., Noguchi, K., Gendo, Y., Ozaki, T., Sonoda, A., Sachi, N., Runtuwene, L. R., Miura, Y., Matsubara, E., Tajima, S., Takasaki, T., Eshita, Y., and Kobayashi, T. (2017) Ribavirin inhibits Zika virus (ZIKV) replication in vitro and suppresses viremia in ZIKV-infected STAT1-deficient mice. *Antiviral Res.* **146**, 1–11

7. Yang, S., Xu, M., Lee, E. M., Gorshkov, K., Shiryaev, S. A., He, S., Sun, W., Cheng, Y. S., Hu, X., Tharappel, A. M., Lu, B., Pinto, A., Farhy, C., Huang, C. T., Zhang, Z., Zhu, W., Wu, Y., Zhou, Y., Song, G., Zhu, H., Shamim, K., Martínez-Romero, C., García-Sastre, A., Preston, R. A., Jayaweera, D. T., Huang, R., Huang, W., Xia, M., Simeonov, A., Ming, G., Qiu, X., Terskikh, A. V., Tang, H., Song, H., and Zheng, W. (2018) Emetine inhibits Zika and Ebola virus infections through two molecular mechanisms: inhibiting viral replication and decreasing viral entry. *Cell Discov. 2018 41*. **4**, 1–14

8. Costa, V. V., Del Sarto, J. L., Rocha, R. F., Silva, F. R., Doria, J. G., Olmo, I. G., Marques, R. E., Queiroz-Junior, C. M., Foureaux, G., Araújo, J. M. S., Cramer, A., Real, A. L. C. V., Ribeiro, L. S., Sardi, S. I., Ferreir, A. J., Machado, F. S., De Oliveira, A. C., Teixeira, A. L., Nakaya, H. I., Souza, D. G., Ribeiro, F. M., and Teixeira, M. M. (2017) N-Methyl-D-Aspartate (NMDA) receptor blockade prevents neuronal death induced by Zika virus infection. *MBio*. 10.1128/MBIO.00350-17/ASSET/D3DFDEB6-42CF-4FA6-A062-ED40AAE0328D/ASSETS/GRAPHIC/MBO0021732890007.JPEG

9. Yuan, S., Chan, J. F. W., den-Haan, H., Chik, K. K. H., Zhang, A. J., Chan, C. C. S., Poon, V. K. M., Yip, C. C. Y., Mak, W. W. N., Zhu, Z., Zou, Z., Tee, K. M., Cai, J. P., Chan, K. H., de la Peña, J., Pérez-Sánchez, H., Cerón-Carrasco, J. P., and Yuen, K. Y. (2017) Structure-based discovery of clinically approved drugs as Zika virus NS2B-NS3 protease inhibitors that potently inhibit Zika virus infection in vitro and in vivo. *Antiviral Res.* **145**, 33–43

10. Pattnaik, A., Palermo, N., Sahoo, B. R., Yuan, Z., Hu, D., Annamalai, A. S., Vu, H. L. X., Correas, I., Prathipati, P. K., Destache, C. J., Li, Q., Osorio, F. A., Pattnaik, A. K., and Xiang, S. hua (2018) Discovery of a non-nucleoside RNA polymerase inhibitor for blocking Zika virus replication through in silico screening. *Antiviral Res.* **151**, 78–86

11. Julander, J. G., Siddharthan, V., Evans, J., Taylor, R., Tolbert, K., Apuli, C., Stewart, J., Collins, P., Gebre, M., Neilson, S., Van Wettere, A., Lee, Y. M., Sheridan, W. P., Morrey, J. D., and Babu, Y. S. (2017) Efficacy of the broad-spectrum antiviral compound BCX4430 against Zika virus in cell culture and in a mouse model. *Antiviral Res.* **137**, 14–22

12. Adcock, R. S., Chu, Y. K., Golden, J. E., and Chung, D. H. (2017) Evaluation of anti-Zika virus activities of broad-spectrum antivirals and NIH clinical collection compounds using a cell-based, high-throughput screen assay. *Antiviral Res.* **138**, 47–56

13. Deng, Y. Q., Zhang, N. N., Li, C. F., Tian, M., Hao, J. N., Xie, X. P., Shi, P. Y., and Qin, C. F. (2016) Adenosine Analog NITD008 Is a Potent Inhibitor of Zika Virus. *Open Forum Infect. Dis.* 10.1093/OFID/OFW175

14. Eyer, L., Nencka, R., Huvarová, I., Palus, M., Alves, M. J., Gould, E. A., De Clercq, E., and Ruzek, D. (2016) Nucleoside Inhibitors of Zika Virus. *J. Infect. Dis.* **214**, 707–711

15. Zmurko, J., Marques, R. E., Schols, D., Verbeken, E., Kaptein, S. J. F., and Neyts, J. (2016) The Viral Polymerase Inhibitor 7-Deaza-2’-C-Methyladenosine Is a Potent Inhibitor of In Vitro Zika Virus Replication and Delays Disease Progression in a Robust Mouse Infection Model. *PLoS Negl. Trop. Dis.* **10**, e0004695

16. Chen, L., Liu, Y., Wang, S., Sun, J., Wang, P., Xin, Q., Zhang, L., Xiao, G., and Wang, W. (2017) Antiviral activity of peptide inhibitors derived from the protein E stem against Japanese encephalitis and Zika viruses. *Antiviral Res.* **141**, 140–149

17. Jackman, J. A., Costa, V. V., Park, S., Real, A. L. C. V., Park, J. H., Cardozo, P. L., Ferhan, A. R., Olmo, I. G., Moreira, T. P., Bambirra, J. L., Queiroz, V. F., Queiroz-Junior, C. M., Foureaux, G., Souza, D. G., Ribeiro, F. M., Yoon, B. K., Wynendaele, E., De Spiegeleer, B., Teixeira, M. M., and Cho, N. J. (2018) Therapeutic treatment of Zika virus infection using a brain-penetrating antiviral peptide. *Nat. Mater. 2018 1711*. **17**, 971–977

18. Shiryaev, S. A., Farhy, C., Pinto, A., Huang, C. T., Simonetti, N., Ngono, A. E., Dewing, A., Shresta, S., Pinkerton, A. B., Cieplak, P., Strongin, A. Y., and Terskikh, A. V. (2017) Characterization of the Zika virus two-component NS2B-NS3 protease and structure-assisted identification of allosteric small-molecule antagonists. *Antiviral Res.* **143**, 218–229

19. Li, C., Deng, Y. Q., Wang, S., Ma, F., Aliyari, R., Huang, X. Y., Zhang, N. N., Watanabe, M., Dong, H. L., Liu, P., Li, X. F., Ye, Q., Tian, M., Hong, S., Fan, J., Zhao, H., Li, L., Vishlaghi, N., Buth, J. E., Au, C., Liu, Y., Lu, N., Du, P., Qin, F. X. F., Zhang, B., Gong, D., Dai, X., Sun, R., Novitch, B. G., Xu, Z., Qin, C. F., and Cheng, G. (2017) 25-Hydroxycholesterol Protects Host against Zika Virus Infection and Its Associated Microcephaly in a Mouse Model. *Immunity*. **46**, 446–456

20. Chen, Y., Li, Z., Pan, P., Lao, Z., Xu, J., Li, Z., Zhan, S., Liu, X., Wu, Y., Wang, W., and Li, G. (2021) Cinnamic acid inhibits Zika virus by inhibiting RdRp activity. *Antiviral Res.* **192**, 105117

21. Coluccia, A., Puxeddu, M., Nalli, M., Wei, C. K., Wu, Y. H., Mastrangelo, E., Elamin, T., Tarantino, D., Bugert, J. J., Schreiner, B., Nolte, J., Schwarze, F., La Regina, G., Lee, J. C., and Silvestri, R. (2020) Discovery of Zika Virus NS2B/NS3 Inhibitors That Prevent Mice from Life-Threatening Infection and Brain Damage. *ACS Med. Chem. Lett.* **11**, 1869–1874

22. Li, Z., Xu, J., Lang, Y., Fan, X., Kuo, L., D’Brant, L., Hu, S., Samrat, S. K., Trudeau, N., Tharappel, A. M., Rugenstein, N., Koetzner, C. A., Zhang, J., Chen, H., Kramer, L. D., Butler, D., Zhang, Q. Y., Zhou, J., and Li, H. (2020) JMX0207, a Niclosamide Derivative with Improved Pharmacokinetics, Suppresses Zika Virus Infection Both in Vitro and in Vivo. *ACS Infect. Dis.* **6**, 2616–2628

23. Li, Z., Sakamuru, S., Huang, R., Brecher, M., Koetzner, C. A., Zhang, J., Chen, H., Qin, C. feng, Zhang, Q. Y., Zhou, J., Kramer, L. D., Xia, M., and Li, H. (2018) Erythrosin B is a potent and broad-spectrum orthosteric inhibitor of the flavivirus NS2B-NS3 protease. *Antiviral Res.* **150**, 217–225

24. Li, Z., Xu, J., Lang, Y., Wu, X., Hu, S., Samrat, S. K., Tharappel, A. M., Kuo, L., Butler, D., Song, Y., Zhang, Q. Y., Zhou, J., and Li, H. (2022) In vitro and in vivo characterization of erythrosin B and derivatives against Zika virus. *Acta Pharm. Sin. B*. **12**, 1662–1670

25. Miao, J., Yuan, H., Rao, J., Zou, J., Yang, K., Peng, G., Cao, S., Chen, H., and Song, Y. (2022) Identification of a small compound that specifically inhibits Zika virus in vitro and in vivo by targeting the NS2B-NS3 protease. *Antiviral Res.* **199**, 105255
