# Supplementary material for: Drugs to limit Zika virus infection and implication for maternal-fetal health
Source: Front Virol. Author manuscript; Available in PMC 2023 Apr 14. (PMC10104533; doi:10.3389/fviro.2022.928599)
Supplement: Supplementary Table 2 [file NIHMS1839512-supplement-Supplementary_Table_2.docx]

**Supplementary Table 2:** A summary of compounds tested *in vitro* with inhibition potential for ZIKV. [N.D. = not determined; EC_50_= half maximal effective concentration; IC_50_= half maximal inhibitory concentration; IC_90_= concentration which shows 90% inhibition]

| **Compound** | **Cell line (anti-ZIKV activity of compounds, ZIKV strain)** | **Mechanistic insight** | **Description of compound** | **Ref.** |
| --- | --- | --- | --- | --- |
| 5-fluorouracil | C6/36 (2 µM, significantly inhibited FSS13025 infection), Huh7 (EC_50_: 14.3 µM, KX197192.1) | N.D. | FDA approved drug | (1, 2) |
| Mycophenolic acid (MPA) | C6/36 (0.5 µM, significantly inhibited FSS13025 infection), Huh-7 (EC_50_: 0.1-1.0 µM, ZIKV MEX_I_7) HeLa (1 µM completely inhibited ZIKV MEX_I_7 infection), JEG3 (1 & 10 µM inhibited ZIKV MEX_I_7 infection), hNSC (1 µM shows inhibition to ZIKV DAK_41525 infection), HAEC (1.6 µM inhibited ZIKV MEX_I_7 infection), Vero (EC_50_ : 0.32-0.77 µM, PLCal_ZV) | N.D. | FDA approved drug | (2–4) |
| Ivermectin | Huh-7 (EC_50_: 1-10 µM, ZIKV MEX_I_7), JEG3 (10 µM, significantly inhibited ZIKV MEX_I_7 infection) | N.D. | FDA approved drug | (4) |
| Digoxin | Vero (IC_50_: 91.82 nM; H/PF/2013; 24.5-784 nM, inhibits MRS infection), Huh-7 (12.25-784 nM, inhibit H/PF/2013 and MRS infection), U251 (98-784 nM, inhibit of H/PF/2013 infection; 196-784 nM, inhibit MRS infection) | Targets Na^+^/K^+^-ATPase. | FDA approved drug | (5) |
| Bortezomib | Vero (IC_50_: 7.67-31.04 µM; PAN2016, R116265, PAN2015, FLR, R103451, PRVABC59, PLCal_ZV, IbH 30656, MEX 2–81 & MR766), Huh-7 (0.1-10 µM, inhibited ZIKV MEX_I_7 infection), hNSC (1 µM ZIKV DAK_41525) | N.D. | FDA approved drug | (4, 6) |
| Daptomycin | Huh-7 (0.1-1 µM, ZIKV MEX_I_7 infection), HeLa (10 µM, reduces ZIKV MEX_I_7 infection), HAECs (16 µM, moderate inhibition of ZIKV MEX_I_7 infection) | N.D. | FDA approved drug | (4) |
| Clomiphene, Alverine, Sertraline | BHK-21 (10 µM, reduces GZ01/2016 infection) | N.D. | FDA approved drug | (7) |
| Nitazoxanide | A549 (EC_50_: 1.48 µM, PRVABC59; 10 or 30 µM reduces SZ-WIV01 infection), hNPCs (considerable reduction in viral titer, PRVABC59), HDF9 (considerable reduction in viral titer, PRVABC59), HPEC (effective as antiviral, PRVABC59), Vero (10 µM, 100-fold reduction in virus particle SZ-WIV01) | Disrupts the interaction between NS2B and NS3 protease. | FDA approved drug | (8, 9) |
| Niclosamide | A549 (EC_50_: 0.48 µM, PRVABC59), HDF9 (considerable reduction in viral titer, PRVABC59), hNPCs (considerable reduction in viral titer, PRVABC59), HPEC (effective as antiviral against PRVABC59), SNB-19 (IC_50_: 0.37 µM, FSS13025; IC_50_: 0.28 µM, PRVABC59), human astrocytes (IC_50_: 0.37 µM, PRVABC59) | Disrupts the interaction between NS2B and NS3 protease. | FDA approved drug | (9, 10) |
| Asunaprevir | U-87 MG (EC_50_: 4.7 µM, PRVABC-59) | Inhibitor of NS2B-NS3 protease (*in vitro* & *in silico*). | FDA approved drug | (11) |
| Simeprevir | U-87 MG (EC_50_: 0.4 µM, PRVABC-59) | Inhibitor of NS2B-NS3 protease (*in vitro* & *in silico*). | FDA approved drug | (11) |
| Lovastatin | Huh-7 (EC_50_: 20.7 µM, KX197192.1) | N.D. | FDA approved drug | (1) |
| Palonosetron | Huh-7 (EC_50_: 16.3 µM, KX197192.1) | N.D. | FDA approved drug | (1) |
| Kitasamycin | Huh-7 (EC_50_: 41.7 µM, KX197192.1) | N.D. | FDA approved drug | (1) |
| 6-azauridine | Huh-7 (EC_50_: 2.3 µM, KX197192.1), Vero (EC_50_: 3.18 µM, MR766; EC_50_: 3.91 µM, PRVABC59) | N.D. | FDA approved drug | (1, 12) |
| Manidipine | Vero (5 µM, 95 % inhibition of H/PF/2013 replication) | N.D. | FDA approved drug | (13) |
| Cilnidipine | Vero (10 µM, shows significant inhibition of H/PF/2013) | N.D. | FDA approved drug | (13) |
| Methotrexate | Vero (IC_50_: 0.245 µM, H/PAN/2016/BEI-259634), hNSCs (IC_50_: 0.334 µM, H/PAN/2016/BEI-259634) | Targets dihydrofolate reductase. | FDA approved drug | (14) |
| Amodiaquine | Vero (EC_50_: 3.07 µM by MTT assay or 4.40 µM by Plaque assay, PLCal_ZV; 10 µM, 85.7 % reduction in PLCal_ZV infection), BHK-21 (10 µM, inhibits GZ01/2016 infection) | N.D. | FDA approved drug | (3, 7) |
| Emricasan | SNB-19 (IC_50_: 0.13-0.1.06 µM, MR766, FSS13025, PRVABC59), HNPCs (shows neuroprotective activity, FSS13025) | Inhibits ZIKV-induced caspase-3 activity. | FDA approved drug | (10) |
| Quinacrine (QC) | Vero (EC_50_: 2.27 µM, MR766) | N.D. | Acridine drug class | (15) |
| Merimepodib | Huh7 (EC_50_: 0.6 µM, MR766) | N.D. | Drug (IMPDH inhibitor) | (16) |
| Mefloquine (MQ) | Vero (EC_50_: 3.95 µM, MR766) | N.D. | FDA approved drug | (15) |
| Lopinavir-ritonavir | Vero (IC_50_: 4.78 µg/ml, PRVABC59), Huh-7 (IC_50_: 3.31 µg/ml, PRVABC59) | Inhibitor of NS2B-NS3 protease (*in vitro* & *in silico*). | FDA approved drug | (17) |
| Azithromycin | Vero (50 mg/L prevented PF13/251013-18 replication) | N.D. | FDA Approved drug | (18) |
| Gemcitabine | RPE (EC_50_: 0.01 µM, FB-GWUH-2016; also inhibits MR766, H/PF/2013 and MRS_OPY_Martinique_PaRi_2015 infection) | Inhibit viral RNA synthesis and protein. Inhibit the activation of cellular caspase. | FDA approved drug | (19) |
| Finasteride | Vero (EC_50_: 9.85 µM, MR766; EC_50_: 26.58 µM, PRVABC59) | N.D. | FDA approved drug | (12) |
| Mevastatin | Vero (EC_50_: 3.42 µM, MR766; EC_50_: 5.05 µM, PRVABC59) | N.D. | Pro-drug | (12) |
| Clofazimine (riminophenazine) | BHK-21(EC_50_: 4.19 μM, RepZIKV_IRES-Neo replicon), Vero (EC_50_: 0.84 μM, ZIKV^BR^) | Inhibitor of RdRp (*in vitro* & *in silico*). | FDA approved drug | (20) |
| Doxycycline | BJ-5ta (20 μM, 50 % of MR766 reduction) | Inhibits viral entry to host cell. Inhibitor of NS2B-NS3 protease (*in vitro* & *in silico*). | FDA approved drug | (21) |
| Amantadine | Vero (IC_50_: 12.25 μg/ml, ZIKV CIET-01; IC_50_: 26.52 μg/ml, MR766; IC_50_: 29.05 μg/ml, ZIKV R103451) | N.D. | FDA approved drug | (22) |
| Rimantadine | Vero (IC_50_: 5.89 μg/ml, ZIKV CIET-01; IC_50_: 8.95 μg/ml, MR766; IC_50_: 4.91 μg/ml, ZIKV R103451) | N.D. | FDA approved drug | (22) |
| Brequinar | Vero (EC_50_: 0.08 μM, MR766; EC_50_: 0.08 μM PRVABC59) | N.D. | NIH Clinical Compound Collection | (12) |
| Suramin | Vero (EC_50_: 39.8 μM, SL1602) | Interferes with viral attachment to host cell and releases the virus particle. | Inhibitor drug | (23) |
| CID 91632869 | Vero (EC_50_: 1 μM, MR766; EC_50_: 2.17 μM, PRVABC59) | N.D. | NIH Clinical Compound Collection | (12) |
| Tizoxanide | Vero (10 μM, completely abolished the SZ-WIV01 in supernatant) | N.D. | Bioactive metabolite (of Nitazoxanide) | (8) |
| 4-aminoquinoline (GSK369796) | Vero (EC_50_: 2.57 µM, MR766) | N.D. | Derivative of amodiaquine | (15) |
| Resiquimod (R848) | Monocytes (1 µM, 30-fold reduction of MR766 RNA) and MDMs (10 µM, 10-fold reduction of MR766 RNA) | Induces expression of viperin (Interferon stimulated gene) that prevents synthesis of viral RNA. | Small molecule  (Toll-like receptor agonist) | (24) |
| MK-591 | NSCs (IC_50_: 3.1 µM, French Polynesian_2013) | Inhibitor of NS2B-NS3 protease (*in vitro* & *in silico*). | Small molecule | (25) |
| JNJ-404 | NSCs (IC_50_: 3.2 µM, French Polynesian_2013) | Inhibitor of NS2B-NS3 protease (*in vitro* & *in silico*). | Small molecule | (25) |
| AV-C | THF (IC_90_: 5.815 µM, PRVABC59) | Targets type I interferon system. | Small molecule | (26) |
| 2′-C-methylated nucleosides (2’-CMG) | Vero (EC_50_: 22. 25 µM, MR766; EC_50_: 2.20 µM, H/PAN/2016/BEI-259634) | N.D. | Nucleoside analog | (27, 28) |
| 2′-C-methylated nucleosides (2’-CMU) | Vero (EC_50_: 45.45 µM, MR766; EC_50_: 4.21 µM, H/PAN/2016/BEI-259634) | N.D. | Nucleoside analog | (27, 28) |
| 2′-C-methylated nucleosides (2’-CMC) | Vero (EC_50_: 10.51 µM, MR766; EC_50_: 0.30 µM, H/PAN/2016/BEI-259634) | N.D. | Nucleoside analog | (27, 28) |
| 2′-C-methylated nucleosides (2’-CMA) | Vero (EC_50_: 5.26 µM, MR766; EC_50_: 0.60 µM, H/PAN/2016/BEI-259634) | N.D. | Nucleoside analog | (27, 28) |
| Obatoclax | RPE (IC_50_: 0.04 µM, FB-GWUH-2016; also inhibits MR766, H/PF/2013 and MRS_OPY_Martinique_PaRi_2015 strain), Vero (EC_50_: 0.13 µM, ZIKV-UbiNanoLuc) | Blocks viral entry to host cell. Inhibits viral RNA and protein synthesis. Inhibits activation of cellular caspase. Prevents ZIKV mediated transcription of antiviral response gene. | Small molecule (Experimental drug) | (19, 29) |
| Saliphenyl-halamide  (SaliPhe) | RPE (IC_50_: 0.05 µM, FB-GWUH-2016; also inhibits MR766, H/PF/2013 and MRS_OPY_Martinique_PaRi_2015 infection) | Inhibits viral RNA and protein synthesis. Inhibits activation of cellular caspase. Prevents ZIKV mediated transcription of antiviral response gene. | NIH Clinical Compound Collection | (19) |
| EGCG | Vero (EC_50_: 21.4 µM, ZIKV^BR^; 25 µM, inhibited 85% entry of MR766) | Blocks viral entry to host cell. | Green tea molecule (polyphenol) | (30) |
| Nanchangmycin | U2OS (IC_50_: 0.128 µM, MEX 2-81), JEG3 (IC_50_: 0.233 µM, MEX 2-81), HBMEC (IC_50_: 0.405 µM, MEX 2-81) | Blocks viral entry to host cell. | Natural product (bioactive compound) | (31) |
| ZINC23845959 | Vero (IC_50_: 2.9 µM, MR766) | Interacts with E protein (*in silico*). | Small molecule | (32) |
| ZINC23400466 | Vero (IC_50_: 5.2 µM, MR766) | Interacts with E protein (*in silico*). | Small molecule | (32) |
| ZINC12415353 | Vero (10 µM, significant protection from MR766) | Interacts with E protein (*in silico*). | Small molecule | (32) |
| F1065-0358 | Vero (IC_50_: 14 µM, nano luciferase ZIKV construct) | Targets conformational rearrangement of E protein (*in silico*). | Small molecule | (33) |
| Gossypol | Vero (IC_50_: 0.21-4.31 µM; PAN2016, R116265, PAN2015, FLR, R103451, PRVABC59, PLCal_ZV, IbH 30656, MEX 2–81 & MR766) | Targets EDIII domain of E protein (*in vitro*). | Natural product | (6) |
| Curcumin | Vero (IC_50_: 5.62-16.57µM; PAN2016, R116265, PAN2015, FLR, R103451, PRVABC59, PLCal_ZV, IbH 30656, MEX 2–81, MR766), HeLa (IC_50_: 1.9µM, HD78788) BHK-21 (5 µM, reduction in the titer of HD78788) | Blocks viral entry to host cell. | Natural product | (6, 34–36) |
| Digitonin | Vero (IC_50_: 3.19-6.52 µM; PAN2016, R116265, PAN2015, FLR, R103451, PRVABC59, PLCal_ZV, IbH 30656, MEX 2–81, MR766) | Blocks viral entry to host cell. | Natural product | (6) |
| Conessine | Vero (IC_50_: 7.18-11.60 µM; PAN2016, R116265, PAN2015, FLR, R103451, PRVABC59, PLCal_ZV, IbH 30656, MEX 2–81 & MR766) | Blocks viral entry to host cell. | Natural product | (6) |
| bis-naphthoquinones | Vero (EC_50_: 0.62-1.38 µM MR766) | N.D. | Naphthoquinones derivative | (37) |
| desethylamodiaquine (DAQ) | Vero (EC_50_: 4.37 µM, PLCal_ZV; EC_50_: 5.59 µM, PRVABC59; EC_50_: 5.21 µM, MR766) | Blocks viral entry to host cell. | Metabolite of amodiaquine drug | (38) |
| N-desethyl-chloroquine (DECQ) | Vero (EC_50_: 7.01 µM, PLCal_ZV; EC_50_: 10.72 µM, PRVABC59; EC_50_: 10.15 µM, MR766) | Blocks viral entry to host cell. | Metabolite of chloroquine drug | (38) |
| Favipiravir (T-705) | Vero (EC_50_: 110.9 µM, SZ01) | N.D. | Nucleoside analog | (39) |
| T-1105 | Vero (EC_50_: 97.5 µM, SZ01) | N.D. | Structure analog of favipiravir (T-705) | (39) |
| 6-methyl-mercaptopurine riboside (6MMPr) | Vero (IC_50_: 24.5 µM, ZIKV PE243), SH-SY5Y (IC_50_: 20.3 µM, ZIKV PE243) | N.D. | Nucleoside analogue | (40) |
| ZINC33683341 | Vero (100 µM, significant inhibition of MR766) | Interacts with E protein (*in silico*) | Small molecule | (41) |
| Harringtonine | Vero (625 nM, 93.8 % inhibition effect on PRVABC59) | Interacts with E protein (*in silico*). Inhibits binding and viral entry to host cell. | Compound from Chinese medicinal herb | (42) |
| Ev37 | Huh-7 (10 µM, reduced 87 % of PRVABC59 infection) | N.D. | Venom peptide from *Euscorpiops validus* | (43) |
| Free-form amino acids precursor (FFAAP) | Vero (ED_90_: 4 mM, PRV59), JEG3 (ED_90_: 2.5 mM, PRV59) | N.D. | Antiviral compound | (44) |
| CLR01 | Vero (IC_50_: 8.2 µM, MR766; IC_50_: 6.7 µM, FBGWUH-2016, IC_50_: 4.2 µM, PRVABC-59) | N.D. | Molecular tweezer | (45) |
| Baicalein | Vero (EC_50_: 0.004-20 µM, PRVABC-59) | Interacts with NS5 (*in silico*). | Flavonoid analog | (46) |
| Baicalin | Vero (EC_50_: 14-31 µM, PRVABC-59) | Blocks viral entry to host cell. Interacts with NS5 (*in silico*). | Flavonoid analog | (46) |
| DMB213 | Huh-7 (EC_50_: 4.6 µM, PLCal_ZV) | Inhibitor of RdRp (*in vitro & in silico*). | Small molecule (Pyridoxine derived) | (47) |
| 4-HPR [N-(4-hydroxyphenyl) retinamide] | Vero (EC_50_: 2.3-3.9 µM, Asian strain/Cook Islands/2014) | Targets interaction between host cell importin α/β1 and ZIKV RdRp. | Synthetic retinoid | (48) |
| F3043-0013 | Vero (EC_50_: 4.8 µM, PLCal_ZV) | Interact with MTase (*in silico*). | Small molecules (life chemicals compounds library) | (49) |
| F0922-0796 | Vero (EC_50_: 12.5 µM, PLCal_ZV) | Interact with MTase (*in silico*). | Small molecules (life chemicals compounds library) | (49) |
| F1609-0442 | Vero (EC_50_: 17.5 µM, PLCal_ZV) | Interact with MTase (*in silico*). | Small molecules (life chemicals compounds library) | (49) |
| F1750-0048 | Vero (EC_50_: 17.6 µM, PLCal_ZV) | Interact with MTase (*in silico*). | Small molecules (life chemicals compounds library) | (49) |
| FV13 (Halogenated Chrysins) | LLC/MK2 (EC_50_: 1.65 µM, SV0010/15) | N.D. | Flavonoid | (50) |
| FV14 (Halogenated Chrysins) | LLC/MK2 (EC_50_: 1.39 µM, SV0010/15) | N.D. | Flavonoid | (50) |
| Compound1 | Vero (EC_50_: 5.95 µM, H/PAN/2016/BEI-259634), hfNSCs (EC_50_: 8.56 µM, H/PAN/2016/BEI-259634) | N.D. | Natural product derivative | (27) |
| PKI 14-22 | HUVEC (IC_50_: 17.75 µM, IbH30656; IC_50_: 22.29 µM, MR766; IC_50_: 34.09 µM, H/FP/2013; IC_50_: 19.19 µM, PRVABC59), Astrocytes (20 µM and 40 µM significantly reduces IbH30656 particle production) | Inhibits protein kinase A (PKA) activation. Interferes with ZIKV RNA and protein synthesis. | Peptide | (51) |
| Cavinafungin | A549 (IC_50_: 150 nM, live reporter virus, pFLZIKV) | Targets signal peptidase to block the cleavage of signal sequence of host and ZIKV protein. | Natural Product | (52) |
| NGI-1 | HEK-293 (EC_50_: 2.2 µM, ZIKV-luciferase; 8 µM, significant inhibition of PRVABC59, P6-740 and PF-13 infection), Huh7 (8 µM, significant reduction in virus particle, PRVABC59) | Inhibits viral RNA synthesis. | Small Molecule | (53) |
| LAS 52154459 | Vero (IC_90_: 5.1 µM, PF13-251013-18) | Targets E-mediated membrane fusion of ZIKV during entry step. | Small molecule | (54) |
| LAS 52509955 | Vero (IC_90_: 10.6 µM, PF13-251013-18) | Targets E-mediated membrane fusion of ZIKV during entry step. | Small molecule | (54) |
| MAC51421886 | Vero (IC_90_: 10.6 µM, PF13-251013-18) | Targets E-mediated membrane fusion of ZIKV during entry step. | Small molecule | (54) |
| LAS 52161573 | Vero (IC_90_: 11.4 µM, PF13-251013-18) | Targets E-mediated membrane fusion of ZIKV during entry step. | Small molecule | (54) |
| LAS 52154463 | Vero (IC_90_: 20.3 µM, PF13-251013-18) | Targets E-mediated membrane fusion of ZIKV during entry step. | Small molecule | (54) |
| LAS 51635112 | Vero (IC_90_: 7.8 µM, PF13-251013-18) | Targets E-mediated membrane fusion of ZIKV during entry step. | Small molecule | (54) |
| LAS 52154474 | Vero (IC_90_: 11.0 µM, PF13-251013-18) | Targets E-mediated membrane fusion of ZIKV during entry step. | Small molecule | (54) |
| NDGA (nordihydroguaiaretic acid) | Vero (IC_50_: 9.1 µM, PA259459) | N.D. | Natural product | (55) |
| M4N (tetra-O-methyl nordihydroguaiaretic acid) | Vero (IC_50_: 5.7 µM, PA259459) | N.D. | Derivative of NDGA | (55) |
| Fatostatin | Vero (35 µM, significantly reduces PA259459 infection) | N.D. | Small molecule | (55) |
| 5-(5-fluorothiophen-2-yl)-2-hydroxy-4-methoxy-N-((3-(trifluoromethyl)phenyl)sulfonyl)benzamide | Huh-7 (EC_50_: 24.3 µM, H/PF/2013) | Inhibitor of RdRp (*in vitro & in silico*). | Non-nucleoside inhibitor | (56) |
| 2,8-bis(trifluoromethyl)quinoline analogs | Vero (EC_50_: 0.8-2 µM, Brazilian ZIKV strain) | N.D. | Mefloquine derivatives | (57) |
| Pentagalloylglucose (PGG) | Vero (IC_50_: 4.1 µM, PRVABC59) | N.D. | Natural products (Polyphenolic compound) | (58) |
| GSK126 | HFF (15 µM, suppress infection of H/PF/2013) | N.D. | EZH2 methyltransferase inhibitor | (59) |
| Bromocriptine | Vero (IC_50_: 13.04 µM, PRVABC59) | Inhibitor of NS2B-NS3 protease (*in vitro* & *in silico*). | FDA approved Drug | (60) |
| Synthetic carbohydrate receptors (SCRs) | Vero (IC_50_: 0.16-12.37 µM, ZIKV RVPs; 8 µM of SCR-15 inhibits PRVABC59 infection), HeLa (IC_50_: 0.24-3.06 µM ZIKV RVPs) | Blocks viral entry to host cell. | Small molecules | (61) |
| 2′-*C*-methyluridine aryloxyl phosphoramidate ProTides | hfNSCs (EC_50_: 2 µM, H/PAN/2016/BEI-259634; EC_50_: 1 µM PRVABC59) | Inhibits RdRp activity (*in vitro)* | Nucleoside analog | (62) |
| 2′-*C*-ethynyluridine aryloxyl phosphoramidate ProTides | hfNSCs (EC_50_: 0.8 µM, H/PAN/2016/BEI-259634; EC_50_: 0.3 µM PRVABC59) | N.D. | Nucleoside analog | (62) |
| 2′-*C*-methyladenosine aryloxyl phosphoramidate ProTides | hfNSCs (EC_50_: 18 µM, H/PAN/2016/BEI-259634; EC_50_: 10 µM PRVABC59) | N.D. | Nucleoside analog | (62) |
| 2′-*C*-methylcitidine aryloxyl phosphoramidate ProTides | hfNSCs (EC_50_: 48 µM PRVABC59) | N.D. | Nucleoside analog | (62) |
| Compound 12a | Vero (EC_50_: 5 µM, MR766) | Interacts with RdRp (*in silico*). | Derivative of Quinoline and 2,6-Diaminopurine | (63) |
| Compound 12b | Vero (EC_50_: 1.5 µM, MR766) | Interacts with RdRp (*in silico*). | Derivative of Quinoline and 2,6-Diaminopurine | (63) |
| Compound 9d | Vero (EC_50_: 2.04 µM, MR766) | N.D. | 2,6-diaminopurine derivatives chemotype | (64) |
| 1ad-af, 1ba-bb, 1bf-bh and 9a | BHK (IC_50_: 4.56-29.98 µM, SMGC) | N.D. | Sinefungin derivatives | (65) |
| NSC135618 | HPECs (EC_50_: 1 µM, PRVABC59), hNPCs (EC_50_: 1.9 µM, PRVABC59), A549 (EC_50_: 1 µM, PRVABC59) | Inhibitor (allosteric) of NS2B-NS3 protease (*in vitro* & *in silico*). | Compound from NCI DPT repository | (66) |
| poly(sodium 4-styrenesulfonate) (PSSNa) | Vero (IC_50_: 8.4-53.5 µg/mL, H/PF/2013), U251 (IC_50_: 8.1-14.4 µg/mL, H/PF/2013), HSF (250 µg/mL suppresses MR766, PRAVABC59, Human/2015/Honduras and Mosquito/1966/Malaysia) | Binds to ZIKV particle and blocks the attachment to host cells. Interact with E protein (*in vitro*). | Polymer | (67) |
| RYL-634 | Huh-7 (EC_50_: 20 nM, ZIKV) | Target Human dihydroorotate dehydrogenase. | Small molecule | (68) |
| Berberine | Vero (IC_50_: 39.06 µM, brazilian ZIKV strain isolated from febrile patient in north east of brazil) | N.D. | Natural Product (isolated from oriental medicinal herbs) | (69) |
| Emodin | Vero (IC_50_: 3.2 µM, brazilian ZIKV strain isolated from febrile patient in north east of brazil) | Blocks viral entry to host cell. | Natural Product (isolated from oriental medicinal herbs) | (69) |
| Pinocembrin | JEG3 (IC_50_: 17.4 µM, PRVABC59), Huh-7 (19.5 µM or 39 µM significantly inhibited PRVABC59 infection) | Inhibits ZIKV RNA and E protein synthesis. | Flavonoid derivative | (70) |
| PF-06409577 | Vero (EC_50_: 2.6 µM, PA259459) | Modulates lipid metabolism of host cell. | Small molecule | (71) |
| Piperlongumine (PL) | HBMEC (IC_50_: 5.086 µmol/L, Z16006), Vero (IC_50_: 3.615 µmol/L, Z16006), HUVEC (IC_50_: 22.359 µmol/L, Z16006) | N.D. | Natural product (amide alkaloid) | (72) |
| 3′-Deoxy-3′-Fluoroadenosine | PS (EC_50_: 1.1 µM, MR766; EC_50_: 1.6 µM, Paraiba_01), HBCA (EC_50_: 4.7 µM, MR766; EC_50_: 4.5 µM, Paraiba_01) | N.D. | Nucleoside analogue | (73) |
| Glycyrrhizic acid (GL) conjugate with amino acid ester | SF268 (IC_50_: 0.09-2.23 µM PRVABC59), A549 (IC_50_: 0.74-1.20 µM, Natal RGN), TE671 (compound13, IC_50_: 0.43µM against ZIKV) | Interacts with MTase (*in silico*). | Derivative of glycyrrhizic acid | (74) |
| 7-deazaadenine ribonucleosides derivatives | Huh-7 (EC_50_: 0.21-26 µM, MP1751) | Inhibitor of RdRp (*in vitro*) | Nucleoside analog | (75) |
| Compound 1 | HEK-293 (IC_50_: 0.75 μM, PRVABC59), Huh-7 (IC_50_: 0.77 μM, NCCP43245; IC_50_: 1.7 μM, H/PF/2013; IC_50_: 5 μM, MR766; IC_50_: 0.69 μM, PRVABC59) | Inhibitor of NS2B-NS3 protease (*in vitro* & *in silico*). | Korea Chemical Bank screening compound | (76) |
| RO8191 (imidazonaphthyridine) | BHK-21(EC_50_: 0.042 μM, RepZIKV_IRES-Neo replicon), Vero (EC_50_: 0.22 μM, ZIKV^BR^) | N.D. | Small molecule (Interferon-like molecule) | (20) |
| Theaflavin | Huh-7 (EC_50_: 8.19 μM, SMGC-1) | Inhibitor of MTase (*in vitro* & *in silico*). | Natural product (polyphenol derived from tea) | (77) |
| Fludarabine | Vero (IC_50_: 0.13 μM, SZ01; IC_50_: 0.19 μM, MR766), BHK-21 (IC_50_: 0.41 μM, SZ01), U251 MG (IC_50_: 0.54 μM, SZ01), MHC3 (IC_50_: 0.71 μM, SZ01) | Impact on viral RNA synthesis. | fluorinated purine analogue | (78) |
| Compounds 22 and trans-14 | A549 (EC_50_: 0.2-1.2 μM; MR766, PRVAVC59, FLR), JEG3 (EC_50_: 0.1-0.6 μM; MR766, PRVAVC59, FLR) | Targets NS4B protein, host pTMD1 domain, RNA replication and assembly. | Derivatives of indole alkaloid | (79) |
| 1,2,3-thiadiazole 30-butyl glycyrrhetinic acid (GA) | SF268 (IC_50_: 0. 50 μM or 0.43 μM; PRVABC59) | Blocks viral entry to host cell. Interact with NS2B-NS3 protease (*in silico*). Inhibits viral protein translation. | Derivative of glycyrrhetinic acid | (80) |
| 3-O-acetyl-30-aminopyridine GA | SF268 (IC_50_: 0.68 μM or 1.00 μM; PRVABC59) | Interact with NS2B-NS3 protease (*in silico*). Inhibits viral protein translation. | Derivative of glycyrrhetinic acid | (80) |
| 3-semicarbazone-30-butyl GA | SF268 (IC_50_: 1.09 μM or 0.45 μM; PRVABC59) | Inhibits viral protein translation. | Derivative of glycyrrhetinic acid | (80) |
| 1,2,3-thiadiazole-30-methyl GA | SF268 (IC_50_: 0.62 μM or 1.94 μM; PRVABC59) | Blocks viral entry to host cell. Inhibits viral protein translation. | Derivative of glycyrrhetinic acid | (80) |
| Destruxins (DTXs) | A549 (10 μM significantly inhibit MR766) | Blocks viral entry or fusion to host cell. Inhibits ZIKV RNA replication and production of NS5. | Peptides (cyclohexadesipeptides analogs) | (81) |
| TCMDC-143406 | Vero (EC_50_: 0.5 μM, PA259459) | Inhibitor of RdRp (*in vitro*). | Antikinetoplastid Compounds | (82) |
| TCMDC-143215 | Vero (EC_50_: 2.6 μM, PA259459) | Inhibitor of RdRp (*in vitro*). | Antikinetoplastid Compounds | (82) |
| Cherylline | Huh7 (EC_50_: 20.3 μM, ZIKV_R2A_), Vero (88 % reduction in viral titer, MR766; 100-fold reduction in viral titer, H/PF/2013) | N.D. | Alkaloid (Amaryllidaceae) | (83) |
| PF-429242 | Vero (35 μM, inhibits PA259459 infection), SK-N-SH ( IC_50_: 13.3 µM, MR766; IC_50_:11.9 µM, H/PF/2013), HeLa (IC_50_: 6.3 µM, MR766; IC_50_: 9.4 µM, H/PF/2013), T98G (IC_50_: 6.9 µM, MR766; IC_50_: 3.5 µM, H/PF/2013), U87MG (IC_50_: 14 µM, MR766; IC_50_: 11.7 µM, H/PF/2013), hMoCD14+-PB (IC_50_: 70.22 µM, MR766; IC_50_: 51.55 µM, H/PF/2013) | N.D. | Small molecule (inhibitor of SREBP) | (55, 84) |
| TDB-2M-ME | Vero, (inhibits 54 % viral infection, ZIKV/Col_2015) | Targets NS3 helicase and cellular DDC and β2 adrenoreceptor (*in silico*). | Dihalogenated Phenolic derivates of L-Tyrosine | (85) |
| TDC-2M-ME | Vero, (inhibits 34.1 % viral infection, ZIKV/Col_2015) | Targets NS3 helicase and cellular DDC and β2 adrenoreceptor (*in silico*). | Dihalogenated Phenolic derivates of L-Tyrosine | (85) |
| Palmitoleate | JAR (100 and 200 μM significantly reduces MR766 infection), HTR-8 (100 and 200 μM significantly reduces MR766 infection) | Protects from ZIKV induced apoptosis and ER stress. | Monounsaturated fatty acid | (86) |
| Labyrinthopeptin A1 | Vero (IC_50_: 0.51-0.99 μM; MR766, PRVABC59, FLR, IBH30656), U87 (IC_50_: 0.5 μM, MR766), JEG3 (IC_50_: 2.5 μM, MR766) | Interacts with E protein. Blocks viral entry to host cells. | Peptide (carbocyclic lantibiotic) | (87) |
| Compound 17 | Huh-7 (EC_50_: 1.25 μM, H/PF/2013) | Inhibitor of NS2B-NS3 protease (*in vitro* & *in silico*). | Derivative of carbazole | (88) |
| Bithiazole derivatives | Huh-7 (IC_50_: 0.51- 46.50 μM, H/PF/2013) | PI4KIIIβ‐targeting inhibitor. | Broad spectrum antiviral agents | (89) |
| Braco-19 | Vero (100 μM, >80-fold reduction in virus growth, MR766) | Inhibits ZIKV genome replication and protein production. Ligand binds to the G-quadruplex. | Small molecule | (90) |
| TMPyP4 | Vero (10 μM, 170-fold reduction in virus growth, MR766) | Inhibits ZIKV genome replication and viral protein synthesis. Ligand binds to the G-quadruplex. | Porphyrin compound | (90) |
| MK-801 | Neuronal cells (10 or 100 μM, reduces ZIKV induced cell, HS-2015-BA-01) | N.D. | NMDR receptor blocker | (91) |
| Ifenprodil | Neuronal cells (0.001-0.01 μM, partially or fully prevents ZIKV induced cell death, HS-2015-BA-01) | N.D. | NMDR receptor blocker | (91) |
| Agmatine sulfate | Neuronal cells (0.4-50 μM, prevents ZIKV induced cell death, HS-2015-BA-01) | N.D. | NMDR receptor blocker | (91) |
| Trehalose | NPCs (100 mM, 10-fold reduction of PRV infection or H/PAN), cortical neuronal cells (100 mM, 10-fold reduction of PRV or H/PAN infection) | N.D. | Disaccharide | (92) |
| Aurintricarboxilic acid | Vero (IC_50_: 13.87 μM, Paraiba/2015; IC_50_: 15.07 μM, MR766; IC_50_: 15.97 μM, IbH30656; IC_50_: 17.55 μM, PRVABC59; IC_50_: 13.92 μM, French Polynesia/2013; IC_50_: 10.50-14.33 μM, Brazil/2015), A549 (IC_50_: 33.33 μM, Paraiba/2015) | N.D. | Polyanionic aromatic compound | (93) |
| Compound 23 | Vero (EC_50_: 9.79 μM, MR766) | Inhibitor of NS2B-NS3 protease (*in vitro* and *in silico*) | Small molecule | (94) |
| Compound 2 | Vero (IC_50_: 33.2 μM, H/PAN/2016/BEI-259634), Huh7.5 (IC_50_: 37.3 μM, H/PAN/2016/BEI-259634) | N.D. | Ribavirin C-nucleoside analog | (95) |
| NCGC00180572 | NPC (IC_50_: 4.4 μM, MR766) | N.D. | Vomitoxin-like compound | (96) |
| 4-(5-phenyl-1,2,4-oxadiazol-3-yl)-N-(pyridin-3-ylmethyl)aniline [5d] | Vero (EC_50_: 1.35 µM, MR766; EC_50_: 2 µM, PRVABC59; EC_50_: 4.3 µM, Brazil/16821) | N.D. | Oxadiazole derivatives | (97) |
| 6-methyl-7-acetylenenyl-7-deazapurine  nucleoside analog | Vero (EC_50_: 3.1 µM, ZG01; EC_50_: 7.7 µM MR766), SNB19 (EC_50_: 6.4 µM, ZG01; EC_50_: 8.9 µM MR766), Huh7 (EC_50_: 5.7 µM, ZG01; EC_50_: 6.1 µM MR766), A549 (EC_50_: 2.8 µM, ZG01; EC_50_: 6.4 µM MR766) | Inhibitor of RdRp (*in vitro*) | Nucleoside analog | (98) |

References:

1. Freitas-Junior, L. H., Pascoalino, B. S., Courtemanche, G., Cordeiro, M. T., and Gil, L. H. V. G. (2016) Zika antiviral chemotherapy: identification of drugs and promising starting points for drug discovery from an FDA-approved library. *F1000Research 2016 52523*. **5**, 2523

2. Dong, S., Kang, S., and Dimopoulos, G. (2019) Identification of anti-flaviviral drugs with mosquitocidal and anti-Zika virus activity in Aedes aegypti. *PLoS Negl. Trop. Dis.* **13**, e0007681

3. Han, Y., Mesplède, T., Xu, H., Quan, Y., and Wainberg, M. A. (2018) The antimalarial drug amodiaquine possesses anti-ZIKA virus activities. *J. Med. Virol.* **90**, 796–802

4. Barrows, N. J., Campos, R. K., Powell, S. T., Prasanth, K. R., Schott-Lerner, G., Soto-Acosta, R., Galarza-Muñoz, G., McGrath, E. L., Urrabaz-Garza, R., Gao, J., Wu, P., Menon, R., Saade, G., Fernandez-Salas, I., Rossi, S. L., Vasilakis, N., Routh, A., Bradrick, S. S., and Garcia-Blanco, M. A. (2016) A Screen of FDA-Approved Drugs for Inhibitors of Zika Virus Infection. *Cell Host Microbe*. **20**, 259–270

5. Guo, J., Jia, X., Liu, Y., Wang, S., Cao, J., Zhang, B., Xiao, G., and Wang, W. (2020) Inhibition of Na+/K+ ATPase blocks Zika virus infection in mice. *Commun. Biol. 2020 31*. **3**, 1–8

6. Gao, Y., Tai, W., Wang, N., Li, X., Jiang, S., Debnath, A. K., Du, L., and Chen, S. (2019) Identification of Novel Natural Products as Effective and Broad-Spectrum Anti-Zika Virus Inhibitors. *Viruses 2019, Vol. 11, Page 1019*. **11**, 1019

7. Li, C., Zhu, X., Ji, X., Quanquin, N., Deng, Y. Q., Tian, M., Aliyari, R., Zuo, X., Yuan, L., Afridi, S. K., Li, X. F., Jung, J. U., Nielsen-Saines, K., Qin, F. X. F., Qin, C. F., Xu, Z., and Cheng, G. (2017) Chloroquine, a FDA-approved Drug, Prevents Zika Virus Infection and its Associated Congenital Microcephaly in Mice. *EBioMedicine*. **24**, 189–194

8. Cao, R. Y., Xu, Y. fen, Zhang, T. H., Yang, J. J., Yuan, Y., Hao, P., Shi, Y., Zhong, J., and Zhong, W. (2017) Pediatric Drug Nitazoxanide: A Potential Choice for Control of Zika. *Open Forum Infect. Dis.* 10.1093/OFID/OFX009

9. Li, Z., Brecher, M., Deng, Y. Q., Zhang, J., Sakamuru, S., Liu, B., Huang, R., Koetzner, C. A., Allen, C. A., Jones, S. A., Chen, H., Zhang, N. N., Tian, M., Gao, F., Lin, Q., Banavali, N., Zhou, J., Boles, N., Xia, M., Kramer, L. D., Qin, C. F., and Li, H. (2017) Existing drugs as broad-spectrum and potent inhibitors for Zika virus by targeting NS2B-NS3 interaction. *Cell Res. 2017 278*. **27**, 1046–1064

10. Xu, M., Lee, E. M., Wen, Z., Cheng, Y., Huang, W. K., Qian, X., Tcw, J., Kouznetsova, J., Ogden, S. C., Hammack, C., Jacob, F., Nguyen, H. N., Itkin, M., Hanna, C., Shinn, P., Allen, C., Michael, S. G., Simeonov, A., Huang, W., Christian, K. M., Goate, A., Brennand, K. J., Huang, R., Xia, M., Ming, G. L., Zheng, W., Song, H., and Tang, H. (2016) Identification of small-molecule inhibitors of Zika virus infection and induced neural cell death via a drug repurposing screen. *Nat. Med. 2016 2210*. **22**, 1101–1107

11. Pathak, N., Kuo, Y. P., Chang, T. Y., Huang, C. T., Hung, H. C., Hsu, J. T. A., Yu, G. Y., and Yang, J. M. (2020) Zika Virus NS3 Protease Pharmacophore Anchor Model and Drug Discovery. *Sci. Reports 2020 101*. **10**, 1–17

12. Adcock, R. S., Chu, Y. K., Golden, J. E., and Chung, D. H. (2017) Evaluation of anti-Zika virus activities of broad-spectrum antivirals and NIH clinical collection compounds using a cell-based, high-throughput screen assay. *Antiviral Res.* **138**, 47–56

13. Wang, S., Liu, Y., Guo, J., Wang, P., Zhang, L., Xiao, G., and Wang, W. (2017) Screening of FDA-Approved Drugs for Inhibitors of Japanese Encephalitis Virus Infection. *J. Virol.* 10.1128/JVI.01055-17/ASSET/FB797C28-EC88-4F1E-BB96-B620E4155FFD/ASSETS/GRAPHIC/ZJV9991830270007.JPEG

14. Beck, S., Zhu, Z., Oliveira, M. F., Smith, D. M., Rich, J. N., Bernatchez, J. A., and Siqueira-Neto, J. L. (2019) Mechanism of Action of Methotrexate Against Zika Virus. *Viruses 2019, Vol. 11, Page 338*. **11**, 338

15. Balasubramanian, A., Teramoto, T., Kulkarni, A. A., Bhattacharjee, A. K., and Padmanabhan, R. (2017) Antiviral activities of selected antimalarials against dengue virus type 2 and Zika virus. *Antiviral Res.* **137**, 141–150

16. Tong, X., Smith, J., Bukreyeva, N., Koma, T., Manning, J. T., Kalkeri, R., Kwong, A. D., and Paessler, S. (2018) Merimepodib, an IMPDH inhibitor, suppresses replication of Zika virus and other emerging viral pathogens. *Antiviral Res.* **149**, 34–40

17. Yuan, S., Chan, J. F. W., den-Haan, H., Chik, K. K. H., Zhang, A. J., Chan, C. C. S., Poon, V. K. M., Yip, C. C. Y., Mak, W. W. N., Zhu, Z., Zou, Z., Tee, K. M., Cai, J. P., Chan, K. H., de la Peña, J., Pérez-Sánchez, H., Cerón-Carrasco, J. P., and Yuen, K. Y. (2017) Structure-based discovery of clinically approved drugs as Zika virus NS2B-NS3 protease inhibitors that potently inhibit Zika virus infection in vitro and in vivo. *Antiviral Res.* **145**, 33–43

18. Azithromycin Inhibits the Replication of Zika Virus [online] https://www.longdom.org/open-access/azithromycin-inhibits-the-replication-of-zika-virus-1948-5964-1000173.pdf (Accessed January 24, 2022)

19. Kuivanen, S., Bespalov, M. M., Nandania, J., Ianevski, A., Velagapudi, V., De Brabander, J. K., Kainov, D. E., and Vapalahti, O. (2017) Obatoclax, saliphenylhalamide and gemcitabine inhibit Zika virus infection in vitro and differentially affect cellular signaling, transcription and metabolism. *Antiviral Res.* **139**, 117–128

20. Discovery of an imidazonaphthyridine and a riminophenazine as potent anti-Zika virus agents through a replicon-based high-throughput screening - ScienceDirect [online] https://www.sciencedirect.com/science/article/abs/pii/S0168170221000952?via%3Dihub (Accessed January 24, 2022)

21. Teoh, T. C., Al-Harbi, S. J., Abdulrahman, A. Y., and Rothan, H. A. (2021) Doxycycline Interferes with Zika Virus Serine Protease and Inhibits Virus Replication in Human Skin Fibroblasts. *Mol. 2021, Vol. 26, Page 4321*. **26**, 4321

22. Arias-Arias, J. L., Vega-Aguilar, F., Picado-Soto, D., Corrales-Aguilar, E., and Loría, G. D. (2021) In Vitro Inhibition of Zika Virus Replication with Amantadine and Rimantadine Hydrochlorides. *Microbiol. Res.* **12**, 727–738

23. Albulescu, I. C., Kovacikova, K., Tas, A., Snijder, E. J., and van Hemert, M. J. (2017) Suramin inhibits Zika virus replication by interfering with virus attachment and release of infectious particles. *Antiviral Res.* **143**, 230–236

24. Vanwalscappel, B., Tada, T., and Landau, N. R. (2018) Toll-like receptor agonist R848 blocks Zika virus replication by inducing the antiviral protein viperin. *Virology*. **522**, 199–208

25. Abrams, R. P. M., Yasgar, A., Teramoto, T., Lee, M. H., Dorjsuren, D., Eastman, R. T., Malik, N., Zakharov, A. V., Li, W., Bachani, M., Brimacombe, K., Steiner, J. P., Hall, M. D., Balasubramanian, A., Jadhav, A., Padmanabhan, R., Simeonov, A., and Nath, A. (2020) Therapeutic candidates for the Zika virus identified by a high-throughput screen for Zika protease inhibitors. *Proc. Natl. Acad. Sci. U. S. A.* **117**, 31365–31375

26. Pryke, K. M., Abraham, J., Sali, T. M., Gall, B. J., Archer, I., Liu, A., Bambina, S., Baird, J., Gough, M., Chakhtoura, M., Haddad, E. K., Kirby, I. T., Nilsen, A., Streblow, D. N., Hirsch, A. J., Smith, J. L., and Defilippis, V. R. (2017) A novel agonist of the trif pathway induces a cellular state refractory to replication of Zika, Chikungunya, and dengue viruses. *MBio*. 10.1128/MBIO.00452-17/SUPPL_FILE/MBO002173291SF8.PDF

27. Bernatchez, J. A., Yang, Z., Coste, M., Li, J., Beck, S., Liu, Y., Clark, A. E., Zhu, Z., Luna, L. A., Sohl, C. D., Purse, B. W., Li, R., and Siqueira-Neto, J. L. (2018) Development and validation of a phenotypic high-content imaging assay for assessing the antiviral activity of small-molecule inhibitors targeting zika virus. *Antimicrob. Agents Chemother.* 10.1128/AAC.00725-18/ASSET/A89ECA21-CE58-4945-A8DF-0DA071C115C8/ASSETS/GRAPHIC/ZAC0101875150007.JPEG

28. Eyer, L., Nencka, R., Huvarová, I., Palus, M., Alves, M. J., Gould, E. A., De Clercq, E., and Ruzek, D. (2016) Nucleoside Inhibitors of Zika Virus. *J. Infect. Dis.* **214**, 707–711

29. Varghese, F. S., Rausalu, K., Hakanen, M., Saul, S., Kümmerer, B. M., Susi, P., Merits, A., and Ahola, T. (2017) Obatoclax inhibits alphavirus membrane fusion by neutralizing the acidic environment of endocytic compartments. *Antimicrob. Agents Chemother.* 10.1128/AAC.02227-16/SUPPL_FILE/ZAC003175951S1.PDF

30. Carneiro, B. M., Batista, M. N., Braga, A. C. S., Nogueira, M. L., and Rahal, P. (2016) The green tea molecule EGCG inhibits Zika virus entry. *Virology*. **496**, 215–218

31. Rausch, K., Hackett, B. A., Weinbren, N. L., Reeder, S. M., Sadovsky, Y., Hunter, C. A., Schultz, D. C., Coyne, C. B., and Cherry, S. (2017) Screening Bioactives Reveals Nanchangmycin as a Broad Spectrum Antiviral Active against Zika Virus. *Cell Rep.* **18**, 804–815

32. Jacobs, A., Rizzo, R. C., Telehany, S. M., Humby, M. S., Dwight McGee, T., and Riley, S. P. (2020) Identification of zika virus inhibitors using homology modeling and similarity-based screening to target glycoprotein e. *Biochemistry*. **59**, 3709–3724

33. Sharma, N., Prosser, O., Kumar, P., Tuplin, A., and Giri, R. (2020) Small molecule inhibitors possibly targeting the rearrangement of Zika virus envelope protein. *Antiviral Res.* **182**, 104876

34. Kim, M., Choi, H., and Kim, Y. B. (2021) Therapeutic targets and biological mechanisms of action of curcumin against Zika virus: In silico and in vitro analyses. *Eur. J. Pharmacol.* **904**, 174144

35. Pacho, M. N., Pugni, E. N., Díaz Sierra, J. B., Morell, M. L., Sepúlveda, C. S., Damonte, E. B., García, C. C., and D’Accorso, N. B. (2021) Antiviral activity against Zika virus of a new formulation of curcumin in poly lactic-co-glycolic acid nanoparticles. *J. Pharm. Pharmacol.* **73**, 357–365

36. Mounce, B. C., Cesaro, T., Carrau, L., Vallet, T., and Vignuzzi, M. (2017) Curcumin inhibits Zika and chikungunya virus infection by inhibiting cell binding. *Antiviral Res.* **142**, 148–157

37. Gonzaga, D. T. G., Gomes, R. S. P., Marra, R. K. F., da Silva, F. C., Gomes, M. W. L., Ferreira, D. F., Santos, R. M. A., Pinto, A. M. V., Ratcliffe, N. A., Cirne-Santos, C. C., Barros, C. S., Ferreira, V. F., and Paixão, I. C. N. P. (2019) Inhibition of Zika Virus Replication by Synthetic Bis-Naphthoquinones. *J. Braz. Chem. Soc.* **30**, 1697–1706

38. Han, Y., Pham, H. T., Xu, H., Quan, Y., and Mesplède, T. (2019) Antimalarial drugs and their metabolites are potent Zika virus inhibitors. *J. Med. Virol.* **91**, 1182–1190

39. Cai, L., Sun, Y., Song, Y., Xu, L., Bei, Z., Zhang, D., Dou, Y., and Wang, H. (2017) Viral polymerase inhibitors T-705 and T-1105 are potential inhibitors of Zika virus replication. *Arch. Virol.* **162**, 2847–2853

40. de Carvalho, O. V., Félix, D. M., de Mendonça, L. R., de Araújo, C. M. C. S., de Oliveira Franca, R. F., Cordeiro, M. T., Silva Júnior, A., and Pena, L. J. (2017) The thiopurine nucleoside analogue 6-methylmercaptopurine riboside (6MMPr) effectively blocks Zika virus replication. *Int. J. Antimicrob. Agents*. **50**, 718–725

41. Fernando, S., Fernando, T., Stefanik, M., Eyer, L., and Ruzek, D. (2016) An Approach for Zika Virus Inhibition Using Homology Structure of the Envelope Protein. *Mol. Biotechnol.* **58**, 801–806

42. Lai, Z. Z., Ho, Y. J., and Lu, J. W. (2020) Harringtonine Inhibits Zika Virus Infection through Multiple Mechanisms. *Mol. 2020, Vol. 25, Page 4082*. **25**, 4082

43. Li, F., Lang, Y., Ji, Z., Xia, Z., Han, Y., Cheng, Y., Liu, G., Sun, F., Zhao, Y., Gao, M., Chen, Z., Wu, Y., Li, W., and Cao, Z. (2019) A scorpion venom peptide Ev37 restricts viral late entry by alkalizing acidic organelles. *J. Biol. Chem.* **294**, 182–194

44. Vasireddi, M., Crum, A., May, H., Katz, D., and Hilliard, J. (2019) A novel antiviral inhibits Zika virus infection while increasing intracellular glutathione biosynthesis in distinct cell culture models. *Antiviral Res.* **161**, 46–52

45. Röcker, A. E., Müller, J. A., Dietzel, E., Harms, M., Krüger, F., Heid, C., Sowislok, A., Riber, C. F., Kupke, A., Lippold, S., von Einem, J., Beer, J., Knöll, B., Becker, S., Schmidt-Chanasit, J., Otto, M., Vapalahti, O., Zelikin, A. N., Bitan, G., Schrader, T., and Münch, J. (2018) The molecular tweezer CLR01 inhibits Ebola and Zika virus infection. *Antiviral Res.* **152**, 26–35

46. Oo, A., Teoh, B. T., Sam, S. S., Bakar, S. A., and Zandi, K. (2018) Baicalein and baicalin as Zika virus inhibitors. *Arch. Virol. 2018 1642*. **164**, 585–593

47. Xu, H. T., Hassounah, S. A., Colby-Germinario, S. P., Oliveira, M., Fogarty, C., Quan, Y., Han, Y., Golubkov, O., Ibanescu, I., Brenner, B., Stranix, B. R., and Wainberg, M. A. (2017) Purification of Zika virus RNA-dependent RNA polymerase and its use to identify small-molecule Zika inhibitors. *J. Antimicrob. Chemother.* **72**, 727–734

48. Wang, C., Yang, S. N. Y., Smith, K., Forwood, J. K., and Jans, D. A. (2017) Nuclear import inhibitor N-(4-hydroxyphenyl) retinamide targets Zika virus (ZIKV) nonstructural protein 5 to inhibit ZIKV infection. *Biochem. Biophys. Res. Commun.* **493**, 1555–1559

49. Stephen, P., Baz, M., Boivin, G., and Lin, S. X. (2016) Structural Insight into NS5 of Zika Virus Leading to the Discovery of MTase Inhibitors. *J. Am. Chem. Soc.* **138**, 16212–16215

50. Suroengrit, A., Yuttithamnon, W., Srivarangkul, P., Pankaew, S., Kingkaew, K., Chavasiri, W., and Boonyasuppayakorn, S. (2017) Halogenated Chrysins Inhibit Dengue and Zika Virus Infectivity. *Sci. Reports 2017 71*. **7**, 1–11

51. Cheng, F., Ramos da Silva, S., Huang, I.-C., Jung, J. U., and Gao, S.-J. (2018) Suppression of Zika Virus Infection and Replication in Endothelial Cells and Astrocytes by PKA Inhibitor PKI 14-22. *J. Virol.* 10.1128/JVI.02019-17/ASSET/7BCF36D1-A94E-4B84-ABDF-69937F5638E6/ASSETS/GRAPHIC/ZJV0041833150010.JPEG

52. Estoppey, D., Lee, C. M., Janoschke, M., Lee, B. H., Wan, K. F., Dong, H., Mathys, P., Filipuzzi, I., Schuhmann, T., Riedl, R., Aust, T., Galuba, O., McAllister, G., Russ, C., Spiess, M., Bouwmeester, T., Bonamy, G. M. C., and Hoepfner, D. (2017) The Natural Product Cavinafungin Selectively Interferes with Zika and Dengue Virus Replication by Inhibition of the Host Signal Peptidase. *Cell Rep.* **19**, 451–460

53. Puschnik, A. S., Marceau, C. D., Ooi, Y. S., Majzoub, K., Rinis, N., Contessa, J. N., and Carette, J. E. (2017) A Small-Molecule Oligosaccharyltransferase Inhibitor with Pan-flaviviral Activity. *Cell Rep.* **21**, 3032–3039

54. Pitts, J., Hsia, C. Y., Lian, W., Wang, J., Pfeil, M. P., Kwiatkowski, N., Li, Z., Jang, J., Gray, N. S., and Yang, P. L. (2019) Identification of small molecule inhibitors targeting the Zika virus envelope protein. *Antiviral Res.* **164**, 147–153

55. Merino-Ramos, T., Jiménez De Oya, N., Saiz, J. C., and Martín-Acebes, M. A. (2017) Antiviral activity of nordihydroguaiaretic acid and its derivative tetra-O-methyl nordihydroguaiaretic acid against West Nile virus and Zika virus. *Antimicrob. Agents Chemother.* 10.1128/AAC.00376-17/ASSET/16651587-A1B4-4379-BC81-D3CB83E977E7/ASSETS/GRAPHIC/ZAC0071763460005.JPEG

56. Gharbi-Ayachi, A., Santhanakrishnan, S., Wong, Y. H., Chan, K. W. K., Tan, S. T., Bates, R. W., Vasudevan, S. G., El Sahili, A., and Lescar, J. (2020) Non-nucleoside Inhibitors of Zika Virus RNA-Dependent RNA Polymerase. *J. Virol.* 10.1128/JVI.00794-20/ASSET/98760A52-BCD9-4655-856C-B37B35403D04/ASSETS/GRAPHIC/JVI.00794-20-F0009.JPEG

57. Barbosa-Lima, G., Moraes, A. M., Araújo, A. da S., da Silva, E. T., de Freitas, C. S., Vieira, Y. R., Marttorelli, A., Neto, J. C., Bozza, P. T., de Souza, M. V. N., and Souza, T. M. L. (2017) 2,8-bis(trifluoromethyl)quinoline analogs show improved anti-Zika virus activity, compared to mefloquine. *Eur. J. Med. Chem.* **127**, 334–340

58. Behrendt, P., Perin, P., Menzel, N., Banda, D., Pfaender, S., Alves, M. P., Thiel, V., Meulemann, P., Colpitts, C. C., Schang, L. M., Vondran, F. W. R., Anggakusuma, Manns, M. P., Steinmann, E., and Pietschmann, T. (2017) Pentagalloylglucose, a highly bioavailable polyphenolic compound present in Cortex moutan, efficiently blocks hepatitis C virus entry. *Antiviral Res.* **147**, 19–28

59. Arbuckle, J. H., Gardina, P. J., Gordon, D. N., Hickman, H. D., Yewdell, J. W., Pierson, T. C., Myers, T. G., and Kristie, T. M. (2017) Inhibitors of the histone methyltransferases EZH2/1 induce a potent antiviral state and suppress infection by diverse viral pathogens. *MBio*. 10.1128/MBIO.01141-17/SUPPL_FILE/MBO004173422ST2.XLSX

60. Chan, J. F. W., Chik, K. K. H., Yuan, S., Yip, C. C. Y., Zhu, Z., Tee, K. M., Tsang, J. O. L., Chan, C. C. S., Poon, V. K. M., Lu, G., Zhang, A. J., Lai, K. K., Chan, K. H., Kao, R. Y. T., and Yuen, K. Y. (2017) Novel antiviral activity and mechanism of bromocriptine as a Zika virus NS2B-NS3 protease inhibitor. *Antiviral Res.* **141**, 29–37

61. Palanichamy, K., Joshi, A., Mehmetoglu-Gurbuz, T., Bravo, M. F., Shlain, M. A., Schiro, F., Naeem, Y., Garg, H., and Braunschweig, A. B. (2019) Anti-Zika Activity of a Library of Synthetic Carbohydrate Receptors. *J. Med. Chem.* **62**, 4110–4119

62. Bernatchez, J. A., Coste, M., Beck, S., Wells, G. A., Luna, L. A., Clark, A. E., Zhu, Z., Hecht, D., Rich, J. N., Sohl, C. D., Purse, B. W., and Siqueira-Neto, J. L. (2019) Activity of Selected Nucleoside Analogue ProTides against Zika Virus in Human Neural Stem Cells. *Viruses 2019, Vol. 11, Page 365*. **11**, 365

63. Kaptein, S. J. F., Vincetti, P., Crespan, E., Rivera, J. I. A., Costantino, G., Maga, G., Neyts, J., and Radi, M. (2018) Identification of Broad-Spectrum Dengue/Zika Virus Replication Inhibitors by Functionalization of Quinoline and 2,6-Diaminopurine Scaffolds. *ChemMedChem*. **13**, 1371–1376

64. Vincetti, P., Kaptein, S. J. F., Costantino, G., Neyts, J., and Radi, M. (2019) Scaffold Morphing Approach to Expand the Toolbox of Broad-Spectrum Antivirals Blocking Dengue/Zika Replication. *ACS Med. Chem. Lett.* **10**, 558–563

65. Tao, Z., Cao, R., Yan, Y., Huang, G., Lv, K., Li, W., Geng, Y., Zhao, L., Wang, A., He, Q., Yang, J., Fan, S., Huang, M., Guo, H., Zhong, W., and Liu, M. (2018) Design, synthesis and in vitro anti-Zika virus evaluation of novel Sinefungin derivatives. *Eur. J. Med. Chem.* **157**, 994–1004

66. Brecher, M., Li, Z., Liu, B., Zhang, J., Koetzner, C. A., Alifarag, A., Jones, S. A., Lin, Q., Kramer, L. D., and Li, H. (2017) A conformational switch high-throughput screening assay and allosteric inhibition of the flavivirus NS2B-NS3 protease. *PLOS Pathog.* **13**, e1006411

67. Botwina, P., Obłoza, M., Szczepanski, A., Szczubiałka, K., Nowakowska, M., and Pyrć, K. (2020) In Vitro Inhibition of Zika Virus Replication with Poly(Sodium 4-Styrenesulfonate). *Viruses 2020, Vol. 12, Page 926*. **12**, 926

68. Yang, Y., Cao, L., Gao, H., Wu, Y., Wang, Y., Fang, F., Lan, T., Lou, Z., and Rao, Y. (2019) Discovery, Optimization, and Target Identification of Novel Potent Broad-Spectrum Antiviral Inhibitors. *J. Med. Chem.* **62**, 4056–4073

69. Batista, M. N., Braga, A. C. S., Fernandes Campos, G. R., Michel Souza, M., de Matos, R. P. A., Zara Lopes, T., Maria Candido, N., Duarte Lima, M. L., Cristina Machado, F., de Andrade, S. T. Q., Bittar, C., Nogueira, M. L., Carneiro, B. M., Mariutti, R. B., Krishnaswamy Arni, R., Freitas Calmon, M., and Rahal, P. (2019) Natural Products Isolated from Oriental Medicinal Herbs Inactivate Zika Virus. *Viruses 2019, Vol. 11, Page 49*. **11**, 49

70. Lee, J. Le, Loe, M. W. C., Lee, R. C. H., and Chu, J. J. H. (2019) Antiviral activity of pinocembrin against Zika virus replication. *Antiviral Res.* **167**, 13–24

71. Jiménez de Oya, N., Blázquez, A.-B., Casas, J., Saiz, J.-C., and Martín-Acebes, M. A. (2018) Direct Activation of Adenosine Monophosphate-Activated Protein Kinase (AMPK) by PF-06409577 Inhibits Flavivirus Infection through Modification of Host Cell Lipid Metabolism. *Antimicrob. Agents Chemother.* 10.1128/AAC.00360-18/ASSET/2820F5EE-3CBE-4708-9C60-EED38C46769D/ASSETS/GRAPHIC/ZAC0071872580005.JPEG

72. Lu, W., Shi, L., Gao, J., Zhu, H., Hua, Y., Cai, J., Wu, X., Wan, C., Zhao, W., and Zhang, B. (2020) Piperlongumine Inhibits Zika Virus Replication In vitro and Promotes Up-Regulation of HO-1 Expression, Suggesting An Implication of Oxidative Stress. *Virol. Sin. 2020 363*. **36**, 510–520

73. Eyer, L., Svoboda, P., Balvan, J., Vicar, T., Raudenská, M., Štefánik, M., Haviernik, J., Huvarová, I., Straková, P., Rudolf, I., Hubálek, Z., Seley-Radtke, K., de Clercq, E., and Růžek, D. (2021) Broad-spectrum antiviral activity of 3′-Deoxy-3′-fluoroadenosine against emerging flaviviruses. *Antimicrob. Agents Chemother.* 10.1128/AAC.01522-20/SUPPL_FILE/AAC.01522-20-S0001.PDF

74. Baltina, L. A., Hour, M. J., Liu, Y. C., Chang, Y. S., Huang, S. H., Lai, H. C., Kondratenko, R. M., Petrova, S. F., Yunusov, M. S., and Lin, C. W. (2021) Antiviral activity of glycyrrhizic acid conjugates with amino acid esters against Zika virus. *Virus Res.* **294**, 198290

75. Milisavljevic, N., Konkolová, E., Kozák, J., Hodek, J., Veselovská, L., Sýkorová, V., Čížek, K., Pohl, R., Eyer, L., Svoboda, P., Růžek, D., Weber, J., Nencka, R., Bouřa, E., and Hocek, M. (2021) Antiviral Activity of 7-Substituted 7-Deazapurine Ribonucleosides, Monophosphate Prodrugs, and Triphoshates against Emerging RNA Viruses. *ACS Infect. Dis.* **7**, 471–478

76. Shin, H. J., Kim, M. H., Lee, J. Y., Hwang, I., Yoon, G. Y., Kim, H. S., Kwon, Y. C., Ahn, D. G., Kim, K. Do, Kim, B. T., Kim, S. J., and Kim, C. (2021) Structure-Based Virtual Screening: Identification of a Novel NS2B-NS3 Protease Inhibitor with Potent Antiviral Activity against Zika and Dengue Viruses. *Microorg. 2021, Vol. 9, Page 545*. **9**, 545

77. Song, W., Zhang, H., Zhang, Y., Chen, Y., Lin, Y., Han, Y., and Jiang, J. (2021) Identification and Characterization of Zika Virus NS5 Methyltransferase Inhibitors. *Front. Cell. Infect. Microbiol.* **11**, 267

78. Gao, C., Wen, C., Li, Z., Lin, S., Gao, S., Ding, H., Zou, P., Xing, Z., and Yu, Y. (2021) Fludarabine Inhibits Infection of Zika Virus, SFTS Phlebovirus, and Enterovirus A71. *Viruses 2021, Vol. 13, Page 774*. **13**, 774

79. Fikatas, A., Vervaeke, P., Meyen, E., Llor, N., Ordeix, S., Boonen, I., Bletsa, M., Kafetzopoulou, L. E., Lemey, P., Amat, M., Pannecouque, C., and Schols, D. (2021) A novel series of indole alkaloid derivatives inhibit dengue and zika virus infection by interference with the viral replication complex. *Antimicrob. Agents Chemother.* 10.1128/AAC.02349-20/SUPPL_FILE/AAC02349-20_SUPP_S1_SEQ4.PDF

80. Baltina, L. A., Lai, H. C., Liu, Y. C., Huang, S. H., Hour, M. J., Baltina, L. A., Nugumanov, T. R., Borisevich, S. S., Khalilov, L. M., Petrova, S. F., Khursan, S. L., and Lin, C. W. (2021) Glycyrrhetinic acid derivatives as Zika virus inhibitors: Synthesis and antiviral activity in vitro. *Bioorg. Med. Chem.* **41**, 116204

81. Yuan, B., Wu, Z., Ji, W., Liu, D., Guo, X., Yang, D., Fan, A., Jia, H., Ma, M., and Lin, W. (2021) Discovery of cyclohexadepsipeptides with anti-Zika virus activities and biosynthesis of the nonproteinogenic building block (3S)-methyl-L-proline. *J. Biol. Chem.* **297**, 100822

82. Sáez-Álvarez, Y., De Oya, N. J., Águila, C. Del, Saiz, J. C., Arias, A., Agudo, R., and Martín-Acebes, M. A. (2021) Novel nonnucleoside inhibitors of Zika virus polymerase identified through the screening of an open library of antikinetoplastid compounds. *Antimicrob. Agents Chemother.* 10.1128/AAC.00894-21/ASSET/F3B540B4-41D7-4474-AAD0-A12D7A4DA1BD/ASSETS/IMAGES/LARGE/AAC.00894-21-F0004.JPG

83. Ka, S., Merindol, N., Sow, A. A., Singh, A., Landelouci, K., Plourde, M. B., Pépin, G., Masi, M., Di Lecce, R., Evidente, A., Seck, M., Berthoux, L., Chatel-Chaix, L., and Desgagné-Penix, I. (2021) Amaryllidaceae alkaloid cherylline inhibits the replication of dengue and Zika viruses. *Antimicrob. Agents Chemother.* 10.1128/AAC.00398-21/SUPPL_FILE/AAC.00398-21-S0001.PDF

84. Raini, S. K., Takamatsu, Y., Dumre, S. P., Urata, S., Mizukami, S., Moi, M. L., Hayasaka, D., Inoue, S., Morita, K., and Ngwe Tun, M. M. (2021) The novel therapeutic target and inhibitory effects of PF-429242 against Zika virus infection. *Antiviral Res.* **192**, 105121

85. Loaiza-Cano, V., Monsalve-Escudero, L. M., Restrepo, M. P., Quintero-Gil, D. C., Pulido Muñoz, S. A., Galeano, E., Zapata, W., and Martinez-Gutierrez, M. (2021) In Vitro and In Silico Anti-Arboviral Activities of Dihalogenated Phenolic Derivates of L-Tyrosine. *Mol. 2021, Vol. 26, Page 3430*. **26**, 3430

86. Muthuraj, P. G., Pattnaik, A., Sahoo, P. K., Islam, M. T., Pattnaik, A. K., Byrareddy, S. N., Hanson, C., Anderson Berry, A., Kachman, S. D., and Natarajan, S. K. (2021) Palmitoleate Protects against Zika Virus-Induced Placental Trophoblast Apoptosis. *Biomed. 2021, Vol. 9, Page 643*. **9**, 643

87. Oeyen, M., Meyen, E., Noppen, S., Claes, S., Doijen, J., Vermeire, K., Süssmuth, R. D., and Schols, D. (2021) Labyrinthopeptin A1 inhibits dengue and Zika virus infection by interfering with the viral phospholipid membrane. *Virology*. **562**, 74–86

88. Rassias, G., Zogali, V., Swarbrick, C. M. D., Ki Chan, K. W., Chan, S. A., Gwee, C. P., Wang, S., Kaplanai, E., Canko, A., Kiousis, D., Lescar, J., Luo, D., Matsoukas, M. T., and Vasudevan, S. G. (2019) Cell-active carbazole derivatives as inhibitors of the zika virus protease. *Eur. J. Med. Chem.* **180**, 536–545

89. Grazia Martina, M., Vicenti, I., Bauer, L., Crespan, E., Rango, E., Boccuto, A., Olivieri, N., Incerti, M., Zwaagstra, M., Allodi, M., Bertoni, S., Dreassi, E., Zazzi, M., van Kuppeveld, F. J. M., Maga, G., and Radi, M. (2021) Bithiazole Inhibitors of Phosphatidylinositol 4-Kinase (PI4KIIIβ) as Broad-Spectrum Antivirals Blocking the Replication of SARS-CoV-2, Zika Virus, and Human Rhinoviruses. *ChemMedChem*. **16**, 3548–3552

90. Majee, P., Pattnaik, A., Sahoo, B. R., Shankar, U., Pattnaik, A. K., Kumar, A., and Nayak, D. (2021) Inhibition of Zika virus replication by G-quadruplex-binding ligands. *Mol. Ther. - Nucleic Acids*. **23**, 691–701

91. Costa, V. V., Del Sarto, J. L., Rocha, R. F., Silva, F. R., Doria, J. G., Olmo, I. G., Marques, R. E., Queiroz-Junior, C. M., Foureaux, G., Araújo, J. M. S., Cramer, A., Real, A. L. C. V., Ribeiro, L. S., Sardi, S. I., Ferreir, A. J., Machado, F. S., De Oliveira, A. C., Teixeira, A. L., Nakaya, H. I., Souza, D. G., Ribeiro, F. M., and Teixeira, M. M. (2017) N-Methyl-D-Aspartate (NMDA) receptor blockade prevents neuronal death induced by Zika virus infection. *MBio*. 10.1128/MBIO.00350-17/ASSET/D3DFDEB6-42CF-4FA6-A062-ED40AAE0328D/ASSETS/GRAPHIC/MBO0021732890007.JPEG

92. Clark, A. E., Zhu, Z., Krach, F., Rich, J. N., Yeo, G. W., and Spector, D. H. (2021) Zika Virus Is Transmitted in Neural Progenitor Cells via Cell-to-Cell Spread, and Infection Is Inhibited by the Autophagy Inducer Trehalose. *J. Virol.* 10.1128/JVI.02024-20/ASSET/0DA99DCC-2A63-4D52-922F-499DEB415F5D/ASSETS/IMAGES/LARGE/JVI.02024-20-F0007.JPG

93. Park, J. G., Ávila-Pérez, G., Madere, F., Hilimire, T. A., Nogales, A., Almazán, F., and Martínez-Sobrido, L. (2019) Potent inhibition of Zika virus replication by aurintricarboxylic acid. *Front. Microbiol.* **10**, 718

94. Mirza, M. U., Alanko, I., Vanmeert, M., Muzzarelli, K. M., Salo-Ahen, O. M. H., Abdullah, I., Kovari, I. A., Claes, S., De Jonghe, S., Schols, D., Schinazi, R. F., Kovari, L. C., Trant, J. F., Ahmad, S., and Froeyen, M. (2022) The discovery of Zika virus NS2B-NS3 inhibitors with antiviral activity via an integrated virtual screening approach. *Eur. J. Pharm. Sci.* **175**, 106220

95. Gonzalez, S., Brzuska, G., Ouarti, A., Gallier, F., Solarte, C., Ferry, A., Uziel, J., Krol, E., and Lubin-Germain, N. (2022) Anti-HCV and Zika activities of ribavirin C-nucleosides analogues. *Bioorg. Med. Chem.* **68**, 116858

96. Dorjsuren, D., Eastman, R. T., Song, M. J., Yasgar, A., Chen, Y., Bharti, K., Zakharov, A. V., Jadhav, A., Ferrer, M., Shi, P. Y., and Simeonov, A. (2022) A platform of assays for the discovery of anti-Zika small-molecules with activity in a 3D-bioprinted outer-blood-retina model. *PLoS One*. **17**, e0261821

97. Nam, S., Na, H. G., Oh, E. H., Jung, E., Lee, Y. H., Jeong, E. J., Ou, Y. Da, Zhou, B., Ahn, S., Shin, J. S., Han, S. B., and Go, Y. Y. (2022) Discovery and synthesis of 1,2,4-oxadiazole derivatives as novel inhibitors of Zika, dengue, Japanese encephalitis, and classical swine fever virus infections. *Arch. Pharm. Res.* **45**, 280–293

98. Yao, G., Yu, J., Lin, C., Zhu, Y., Duan, A., Li, M., Yuan, J., and Zhang, J. (2022) Design, synthesis, and biological evaluation of novel 2′-methyl-2′-fluoro-6-methyl-7-alkynyl-7-deazapurine nucleoside analogs as anti-Zika virus agents. *Eur. J. Med. Chem.* **234**, 114275
